# Appetite and dietary intake endpoints in cancer cachexia clinical trials: Systematic Review 2 of the cachexia endpoints series

**DOI:** 10.1002/jcsm.13434

**Published:** 2024-02-11

**Authors:** Ola Magne Vagnildhaug, Trude R. Balstad, Inger Ottestad, Asta Bye, Christine Greil, Jann Arends, Vickie Baracos, Leo R. Brown, Olav F. Dajani, Ross D. Dolan, Marie Fallon, Eilidh Fraser, Aleksandra Grzyb, Marianne J. Hjermstad, Gunnhild Jakobsen, Stein Kaasa, James McDonald, Iain Philips, Judith Sayers, Melanie R. Simpson, Mariana S. Sousa, Richard J.E. Skipworth, Barry J.A. Laird, Tora S. Solheim, the Cancer Cachexia Endpoints Working Group

**Affiliations:** ^1^ Department of Clinical and Molecular Medicine, Faculty of Medicine and Health Sciences Norwegian University of Science and Technology (NTNU) Trondheim Norway; ^2^ Cancer Clinic, St. Olavs Hospital Trondheim University Hospital Trondheim Norway; ^3^ Department of Clinical Medicine, Clinical Nutrition Research Group UiT The Arctic University of Norway Tromsø Norway; ^4^ Department of Nutrition, Institute of Basic Medical Sciences, Faculty of Medicine University of Oslo Oslo Norway; ^5^ The Clinical Nutrition Outpatient Clinic, Section of Clinical Nutrition, Department of Clinical Service, Division of Cancer Medicine Oslo University Hospital Oslo Norway; ^6^ Regional Advisory Unit for Palliative Care, Department of Oncology, Oslo University Hospital University of Oslo Oslo Norway; ^7^ European Palliative Care Research Centre (PRC), Department of Oncology, Oslo University Hospital and Institute of Clinical Medicine University of Oslo Oslo Norway; ^8^ Department of Nursing and Health Promotion, Faculty of Health Sciences OsloMet—Oslo Metropolitan University Oslo Norway; ^9^ Department of Medicine I, Medical Center—University of Freiburg, Faculty of Medicine University of Freiburg Freiburg im Breisgau Germany; ^10^ Department of Oncology University of Alberta Edmonton Alberta Canada; ^11^ Clinical Surgery University of Edinburgh, Royal Infirmary of Edinburgh Edinburgh UK; ^12^ Academic Unit of Surgery University of Glasgow, Glasgow Royal Infirmary Glasgow UK; ^13^ Edinburgh Cancer Research Centre University of Edinburgh Edinburgh UK; ^14^ Department of Public Health and Nursing, Faculty of Medicine and Health Sciences Norwegian University of Science and Technology (NTNU) Trondheim Norway; ^15^ Institute of Genetics and Cancer University of Edinburgh Edinburgh UK; ^16^ St Columba's Hospice Edinburgh UK; ^17^ Improving Palliative, Aged and Chronic Care through Clinical Research and Translation (IMPACCT) University of Technology Sydney Sydney New South Wales Australia

**Keywords:** appetite, cachexia, cancer, dietary intake, endpoints, outcomes, trials

## Abstract

There is no consensus on the optimal endpoint(s) in cancer cachexia trials. Endpoint variation is an obstacle when comparing interventions and their clinical value. The aim of this systematic review was to summarize and evaluate endpoints used to assess appetite and dietary intake in cancer cachexia clinical trials. A search for studies published from 1 January 1990 until 2 June 2021 was conducted using MEDLINE, Embase and Cochrane Central Register of Controlled Trials. Eligible studies examined cancer cachexia treatment versus a comparator in adults with assessments of appetite and/or dietary intake as study endpoints, a sample size ≥40 and an intervention lasting ≥14 days. Reporting was in line with PRISMA guidance, and a protocol was published in PROSPERO (2022 CRD42022276710). This review is part of a series of systematic reviews examining cachexia endpoints. Of the 5975 articles identified, 116 were eligible for the wider review series and 80 specifically examined endpoints of appetite (65 studies) and/or dietary intake (21 studies). Six trials assessed both appetite and dietary intake. Appetite was the primary outcome in 15 trials and dietary intake in 7 trials. Median sample size was 101 patients (range 40–628). Forty‐nine studies included multiple primary tumour sites, while 31 studies involved single primary tumour sites (15 gastrointestinal, 7 lung, 7 head and neck and 2 female reproductive organs). The most frequently reported appetite endpoints were visual analogue scale (VAS) and numerical rating scale (NRS) (40%). The appetite item from the European Organisation for Research and Treatment of Cancer Quality of Life Questionnaire (EORTC QLQ) C30/C15 PAL (38%) and the appetite question from North Central Cancer Treatment Group anorexia questionnaire (17%) were also frequently applied. Of the studies that assessed dietary intake, 13 (62%) used food records (prospective registrations) and 10 (48%) used retrospective methods (24‐h recall or dietary history). For VAS/NRS, a mean change of 1.3 corresponded to Hedge's *g* of 0.5 and can be considered a moderate change. For food records, a mean change of 231 kcal/day or 11 g of protein/day corresponded to a moderate change. Choice of endpoint in cachexia trials will depend on factors pertinent to the trial to be conducted. Nevertheless, from trials assessed and available literature, NRS or EORTC QLQ C30/C15 PAL seems suitable for appetite assessments. Appetite and dietary intake endpoints are rarely used as primary outcomes in cancer cachexia. Dietary intake assessments were used mainly to monitor compliance and are not validated in cachexia populations. Given the importance to cachexia studies, dietary intake endpoints must be validated before they are used as endpoints in clinical trials.

## Introduction

Loss of appetite is a critical phenotypic feature of cancer cachexia and, combined with reduced dietary intake, drives weight loss.^1^ The underlying biology of this is complex and attributable to disturbances of homeostatic mechanisms involved in the regulation of energy balance.^2^ The hypothalamus is the key regulator of appetite and can be modulated by several factors including inflammatory cytokines, hormones, neurotransmitters, and sympathetic nerves and vagal afferent fibres.^3^

Appetite loss is associated with impaired quality of life (QoL) in patients with cancer^4^ and contributes to loss of muscle mass, decline in physical function and increased mortality as part of the cachexia syndrome.^5^ Targeting appetite loss has been advocated as a therapeutic strategy to modify this maladaptive response to cancer and to improve QoL. Consequently, clinical trials have for decades aimed to improve appetite and thereby increase dietary intake and body weight in patients at risk of, or suffering from, cachexia. Although improving appetite may not always result in increased body weight,^6^ it remains a valuable treatment objective, given its high prevalence and negative impact on patient QoL. One challenge in assessing appetite is that it can represent several symptoms including early satiety and a reduced desire to eat. This is often compounded by other nutritional impact symptoms (NIS) that may inhibit appetite and induce premature cessation of food intake.^7^ Examples of NIS are alterations in taste and smell, dry mouth, pain and emotional status, and this means that dietary intake is not dependent on appetite alone. Many patients will also force themselves to eat despite a poor appetite. Thus, to fully understand why and how weight loss occurs, comprehensive assessments of appetite, other NIS, and energy and protein intake are necessary.

Currently, there are a multitude of methods for appetite and dietary intake assessment that may be relevant in cancer cachexia trials, and there is no consensus on which are preferable endpoints. Examples include patient‐reported outcome measures (PROMs), using various scales and time frames, and assessment of dietary intake through different methods of recall or prospective registrations.

This breadth of endpoints poses a significant obstacle in comparing interventions and understanding whether an intervention has clinical value.^8^ The ideal endpoint should be meaningful for patients and healthcare professionals alike, reflect the mechanism of action of the intervention being tested, be easy to measure, and be sensitive and specific.^9^, ^10^ Achieving alignment on the assessment of appetite and dietary intake endpoints in cancer cachexia trials is fundamental for successful research outcomes, for comparing results between studies and for conducting meta‐analyses. The first step is to outline and evaluate the endpoints used in existing cachexia trials. This process is necessary for developing a standardized approach to nutritional endpoint selection.

Therefore, the aim of this systematic review is to summarize and evaluate the endpoints employed for assessment of appetite and dietary intake in clinical trials targeting cancer cachexia and to present a comprehensive overview of endpoints used, frequencies, results, and estimates of effect size and required sample size.

## Methods

This systematic review is aligned with the Preferred Reporting Items for Systematic Reviews and Meta‐Analyses (PRISMA) statement.^11^ Covidence systematic review software (Veritas Health Innovation, Melbourne, Australia) was used to streamline the review process. The protocol is registered in the PROSPERO database (CRD42022276710 [http://www.crd.york.ac.uk/PROSPERO]).

### Search strategy

The search for studies published from 1 January 1990 until 2 June 2021 was conducted by a research librarian using the databases MEDLINE (Ovid), Embase (Ovid) and Cochrane Central Register of Controlled Trials (see *Appendix* *S1* for search strategy).

### Study selection and data extraction

Eligible studies were controlled and examined interventions aiming to treat or attenuate cachexia in adult patients (>18 years) with cancer. Studies using pharmacological, nutritional, exercise and/or behavioural interventions were included, and there were no restrictions concerning the comparator(s).

Studies including endpoints assessing appetite and dietary intake were included if the outcome measure was clearly described, and the results were either presented numerically or the statistical significance of a difference between treatment arms was described. Studies using dietary intake outcome measures were included if they presented data on protein intake (g/day, g/kg/day) and/or energy intake (kcal/day, kcal/kg/day, % of estimated needs). Endpoints with a composite score based on additional items not directly related to dietary intake or appetite (e.g., Functional Assessment of Anorexia/Cachexia Therapy Anorexia/Cachexia Subscale [FAACT A/CS]^12^ and Patient‐Generated Subjective Global Assessment [PG‐SGA] global score)^13^ were not eligible unless they explicitly presented results pertaining to the appetite‐related items. Studies using both single items and studies using formalized questionnaires such as the European Organisation for Research and Treatment of Cancer Quality of Life Questionnaire (EORTC QLQ) C30, Edmonton Symptom Assessment Scale (ESAS) questionnaire or the MD Anderson Symptom Inventory (MDASI) with appetite as one of the several scales/items were included. Studies reporting other NIS (e.g., dysphagia, constipation, pain, and alterations of taste or smell), and anorexia only as an adverse event using the Common Terminology Criteria for Adverse Events (CTCAE), were excluded. Studies with <40 patients and/or an intervention duration shorter than 14 days were excluded. Eligible studies had to be published in full‐text format and written in English.

Using Covidence software, two independent reviewers (OFD and BJAL) performed a title‐based selection process for all identified trials. Another pair of independent reviewers (TSS and BJAL) reviewed and selected studies based on their abstracts. Any eligibility uncertainties were resolved through discussions to reach a consensus.

A data extraction table was developed, pilot‐tested and refined within the review group before use. Two independent review authors extracted the data. This systematic review is part of a comprehensive review collaboration covering six main groups of endpoints in cachexia: body composition, oncology, physical function, QoL, biomarkers and nutrition. Because most controlled trials in cachexia explore multiple endpoints, all papers were divided among the review teams, and each team extracted data for all endpoints.

### Assessing risk of bias

Four different reviewers (JM, JS, OFD and BJAL) assessed the methodological quality of each study using the modified Downs and Black Checklist.^14^ The tool assesses among other criteria, study design, external and internal validity, whether spread was reported and if outcome was defined (see *Appendix* *S2* for scoring details).

### Endpoints and data analysis

Data were reported narratively, describing the diversity and frequencies of endpoints. Concordance between endpoints of appetite and dietary intake and between appetite/dietary intake and weight loss was evaluated, whenever possible.

Where the mean difference in change of the endpoint (baseline to post‐intervention) and standard deviation of the difference (or standard error of the mean change) were reported, effect size was calculated using Hedge's *g*. Additionally, an estimate of what constituted a small, medium and large effect size was presented for each selected instrument based on the pooled standard deviation from two or more studies. An estimate of needed sample size to detect a small, medium and large effect size was calculated using the normal approximation to the two‐sample *t* test for independent samples.

## Results

**Figure 1:**
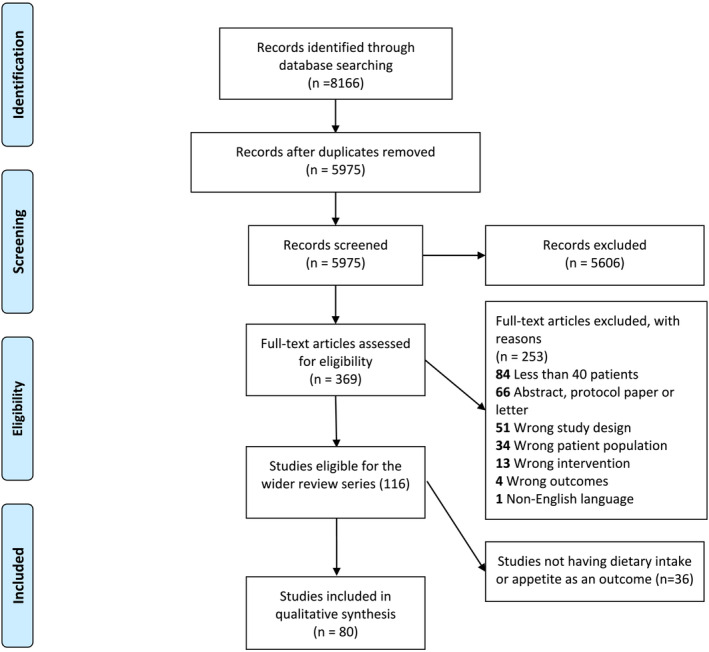
PRISMA flow chart.

*Figure* *1* shows the flow chart for study selection. The literature search resulted in 5975 studies, and following the title and abstract screening, 369 articles were fully reviewed, 116 articles were eligible for the wider review series and 80 articles were eligible for this specific review.

*Table* *1* shows the key characteristics of eligible trials. The sample size ranged from 40 to 628 with a median of 101. Forty‐nine studies included multiple primary tumour sites, while 31 studies focused on single primary tumour sites (15 gastrointestinal [GI], 7 lung, 7 head and neck and 2 female reproductive organs). Pharmacological interventions were used in 43 (54%) studies, nutritional interventions in 25 (31%) studies, multimodal interventions in 9 (11%) studies and behavioural/exercise interventions in 3 (4%) studies.

**Table 1:** Key characteristics of eligible studies

First author	Year	Study type	*N* ^a^	Quality	Cancer type	Cancer treatment	Cachexia inclusion criteria	Intervention	Comparator	Primary outcome	Nutrition outcome
Kardinal^15^	1990	Double‐blind RCT	293	8	Other, GI, lung	No, chemotherapy/immunotherapy and/or radiotherapy	WL > 5 lb in 2 months or intake <20 kcal/kg BW/day	Cyproheptadine 8 mg t.i.d.	Placebo	BW	NCCTG (appetite questions)
Loprinzi^16^	1990	Double‐blind RCT	133	9	GI, lung, other	60% received chemotherapy	WL > 5 lb in 2 months or intake <20 kcal/kg	MA 800 mg/day	Placebo	BW	NCCTG (appetite questions)
Feliu^17^	1992	Double‐blind RCT	150	5	Lung, other, gastric	No tumour‐directed treatment	WL > 10% and/or appetite <5 NRS	MA	Placebo	BW and appetite	Appetite VAS (SSA)
Downer^18^	1993	Double‐blind RCT	60	1	Other, lung, mesothelioma	2/3 received chemotherapy	Loss of appetite, not further described	MPA	Placebo	Not defined	Appetite VAS
Loprinzi^19^	1993	RCT	342	8	Lung, GI, other	No, chemotherapy and/or radiotherapy in palliative intent	WL > 5 lb in 2 months or intake <20 kcal/kg	(1) MA 1280 mg, (2) 800 mg or (3) 400 mg/day	(4) MA 160 mg/day	BW	NCCTG (appetite questions)
Ovesen^20^	1993	Open‐label RCT	137	8	Ovarian, lung, breast	Chemotherapy	Life expectancy ≥2 months, ECOG ≥ 2	Dietary counselling and multivitamin mineral tablet	Standard care Including one daily multivitamin–mineral tablet	BW and QoL	Food records
Lai^21^	1994	RCT	52	5	Cervical, endometrial, colorectal	Radiotherapy	Experiencing anorexia during RT	MA 160 mg/day or prednisolone 30 mg/day	Placebo	Not defined	Appetite study‐specific score
Goldberg^22^	1995	Double‐blind RCT	70	8	Lung, GI, other		WL > 5 lb in 2 months or intake <20 kcal/kg	Pentoxifylline 400 mg t.i.d.	Placebo	BW	NCCTG (appetite questions)
Chen^23^	1996	RCT	129	8	H&N	Radiotherapy	NA	MA 40 mg q.i.d. or cisapride 5 mg t.i.d.	Placebo	BW and appetite	Scored by HCP
Gebbia^24^	1996	RCT	122	6	Lung, H&N, other	Refractory to prior chemotherapy	WL > 5%	MA 160 mg/day	MA 320 mg/day	NA	Appetite Symptom Distress Scale
Simons^25^	1996	Double‐blind RCT	206	7	NSCLC, GI, other	Concurrent chemotherapy 12% in both groups	NA	MPA	Placebo	Appetite, weight and QoL	Appetite NRS, EORTC QLQ C‐30 (appetite item)
Beller^26^	1997	Double‐blind RCT	240	4	GI, lung, other	50% received chemotherapy	BW ≥ 5% below ideal or WL > 5%	(1) MA high dose	(2) MA low dose (3) Placebo	QoL, combined	Appetite VAS (LASA)
Catalina^27^	1998	Double‐blind RCT	150	5	Lung, other, colorectal	Majority without chemotherapy	WL > 5% during illness	(1) MA low dose (2) MA high‐dose group	(3) Placebo group	NA	Appetite VAS
De Conno^28^	1998	RCT	42	6	Lung, other, GI	BSC	Diminished or absent appetite	MA	Placebo	Appetite	NRS appetite
Simons^29^	1998	Double‐blind RCT	54	6	NSCLC, GI, other	No chemotherapy	NA	MPA 500 mg b.i.d.	Placebo	Energy and protein intake, body composition	Dietary history
Loprinzi^30^	1999	RCT	496	8	Lung, GI, other		WL > 5 lb in 2 months or intake <20 kcal/kg	(1) MA 800 mg/day (2) Dexamethasone 0.75 mg q.i.d.	(3) Fluoxymesterone 10 mg b.i.d.	Appetite	NCCTG (appetite questions)
McMillan^31^	1999	Double‐blind RCT	73	7	GI (67% pancreatic)	BSC	WL > 5%	MA + ibuprofen	MA + placebo	BW	Appetite VAS, EORTC QLQ C‐30 (appetite item)
Westman^32^	1999	Double‐blind RCT	255	7	Colorectal, lung, other	Treatment with palliative intent	Progressive, symptomatic cancer, preferably (not necessarily) anorexia and/or WL	MA	Placebo	QoL	EORTC QLQ C‐30 (appetite item)
Erkurt^33^	2000	RCT	100	5	Lung, H&N, other	Chemotherapy and/or radiotherapy	Increasing anorexia, smell and taste change and WL due to RT	MA 480 mg/day	Placebo	BW	Scored by HCP
Jatoi^34^	2002	RCT	469	10	Lung, GI, other	70% received chemotherapy	WL 5 lb in 2 months or intake <20 kcal/kg	(1) MA 800 mg/day + dronabinol 5 mg/day (2) Dronabinol 5 mg/day + placebo	(3) MA 800 mg/day + placebo	BW and appetite	NCCTG (appetite questions)
Persson^35^	2002	Open‐label RCT	137	6	Colorectal, gastric	No, chemotherapy and/or radiotherapy in palliative or curative intent	NA	(1) Individual dietary and psychological support (IS) (2) IS + group rehabilitation (GR)	(3) SOC (4) GR	Not defined	EORTC QLQ C‐30 (appetite item)
Ulutin^36^	2002	RCT	119	9	NSCLC	None	WL > 10% in 6 months	MA 160 mg/day	MA 320 mg/day	BW and appetite	Symptom Distress Scale
Bruera^37^	2003	Double‐blind RCT	91	7	Other, GI, urogenital	Palliative treatment	Anorexia (>3 on VAS) plus WL > 5%	Fish oil capsules (EPA, DHA, vitamin E)	Placebo	Appetite	Appetite VAS, food records
Fearon^38^	2003	Double‐blind RCT	200	8	Pancreatic	No previous treatment in the last 4 weeks. Ongoing treatment in trial not described	>5% WL in 6 months	Protein and energy‐dense *n*‐3 PUFA‐enriched ONS	Oral supplement (without *n*‐3 PUFAs)	BW	Food records
Jatoi^39^	2004	Double‐blind RCT	421	8	Other, lung, GI	With/without chemotherapy	WL > 5 lb in 2 months or intake <20 kcal/kg BW/day	EPA	MA	BW	NCCTG (appetite questions), FAACT (appetite question)
Lundholm^40^	2004	Open‐label RCT	309	5	Other, colorectal, gastric	No antitumour treatment, palliative treatment	WL 3–5% over 3 months, ongoing WL due to disease or no tumour therapy available	COX inhibitor, EPO, dietary counselling and total PN (TPN)	COX inhibitor, EPO	BW, energy and protein intake, body composition, exercise capacity	Food records
Bauer^41^	2005	Double‐blind RCT	200	8	Pancreatic	Surgery, chemotherapy or radiotherapy	WL > 5% in the previous 6 months	ONS with EPA	Isocaloric ONS, 2 cans a day	Body composition, energy and protein intake, QoL	Food records
Dias^42^	2005	Non‐RCT Grouped according to nutritional needs	64	1	H&N	Radiotherapy	Not described	Dietary counselling for a pureed food	Group 1: EN Group 2: Oral diet enriched with alimentary supplement	Energy and protein intake	24‐h recall
Ravasco^43^	2005	RCT	75	7	H&N	Preoperative radiochemotherapy	NA	(1) Dietary counselling (2) ONS	(3) Intake ad lib	Energy and protein intake	EORTC QLQ C‐30 (appetite item), dietary history, 24‐h recall
Fearon^44^	2006	Double‐blind RCT	518	8	GI, lung	>4 weeks after chemotherapy/radiotherapy or surgery	≥5% of pre‐illness stable weight	(1) EPA 2 g (2) EPA 4 g	(3) Placebo	BW	EORTC QLQ C‐30 (appetite item)
Strasser^45^	2006	Double‐blind RCT	243	8	GI, urogenital, other	50% received chemotherapy	WL > 5% in 6 months	(1) THC + CBD	(2) THC (3) Placebo	Appetite	Appetite VAS
Jatoi^46^	2007	Double‐blind RCT	63	7	Lung, GI, other	No, chemotherapy and/or radiotherapy	WL > 5 lb in 2 months or intake <20 kcal/kg BW/day	Etanercept 25 mg twice weekly	Placebo	BW	NCCTG (appetite questions)
Beijer^47^	2009	Open‐label RCT	100	8	Lung, other, colon	No curative treatment, details not available	Incurable cancer, life expectancy 1–6 months, WHO PS ≤ 2, one of the following: Fatigue, WL > 5% in the last 6 months, anorexia	Weekly ATP infusion, 8–10 h, dose up to 50 μg/kg/min	Standard nutritional advice	BW, energy and protein intake, body composition, survival	Food records
Jatoi^48^	2009	Double‐blind RCT	61	7	NSCLC	>3 weeks after chemotherapy/radiotherapy or surgery	NA	Infliximab	Placebo	BW	NCCTG (appetite questions)
Mantovani^49^	2010	RCT	332	7	Lung, breast, colorectal	Concomitant palliative chemotherapy in ~80%	WL > 5% of ideal or pre‐illness in the previous 3 months	(1) MPA or MA (2) EPA‐enriched ONS (3) l‐carnitine (4) Thalidomide	(5) Combination: MPA or MA + EPA‐enriched ONS + l‐carnitine + thalidomide	LBM, REE, fatigue	Appetite VAS
Navari^50^	2010	Open‐label RCT	80	7	Lung, GI	>4 weeks after chemotherapy/radiotherapy or surgery	WL ≥ 5% and appetite loss (not defined)	MA 800 mg/day + olanzapine 5 mg/day	MA 800 mg/day	BW and appetite	Appetite VAS (MDASI)
Baldwin^51^	2011	Open‐label RCT	358	8	Colorectal, other, lung	Palliative chemotherapy	Any WL in the last 3 months	(1) Dietary counselling (increase intake 600 kcal/day) (2) Supplement (3) Dietary counselling + supplement	(4) SOC	OS	Appetite EORTC QLQ C‐30 (appetite item)
Silander^52^	2011	RCT	134	6	H&N	Radiochemotherapy or surgery and adjuvant radiotherapy in curative intent	WL > 10% during the last 6 months	EN	SOC	Not defined	EORTC QLQ C‐30 (appetite item)
Wen^53^	2012	RCT	102	5	Other, lung, gastric/breast	Treatment with palliative intent	WL > 5% of pre‐illness or ideal BW	MA + thalidomide	MA	BW, fatigue, EORTC QLQ and safety	Appetite NRS
Maccio^54^	2012	RCT	144	8	Ovarian, endometrial, cervical	Palliative chemotherapy	WL ≥ 5% in 3 months	Lipoic acid + carbocysteine + l‐carnitine + celecoxib + MA	MA	LBM	Appetite VAS
Madeddu^55^	2012	RCT	60	7	Other, H&N, lung	Concomitant palliative chemotherapy in ~80%	WL ≥ 5% of ideal or pre‐illness BW in the previous 6 months (associated with proinflammatory cytokines and/or CRP)	l‐carnitine + celecoxib + MA	l‐carnitine + celecoxib	LBM and total daily physical activity	Appetite VAS
Del Fabbro^56^	2013	Double‐blind RCT	73	10	GI, lung	NA	Appetite loss ≥4 (ESAS) and WL ≥ 5% over 6 months	Melatonin	Placebo	Appetite	Appetite NRS (ESAS)
Dobrila‐Dintinjana^57^	2013	Non‐RCT with historical controls	628	7	Colorectal	Chemotherapy	NA	Dietary counselling + MA and EPA‐enriched ONS	SOC	Nutritional status (not defined), survival	Appetite NRS
Kanat^58^	2013	RCT	62	8	Lung, other, pancreatic	No, chemotherapy/hormone therapy and/or radiotherapy in palliative or curative intent	WL > 5% over 3 months	MA + meloxicam	(1) MA + meloxicam + EPA‐enriched supplement (2) EPA + meloxicam	BW and LBM	Appetite VAS
Bourdel‐Marchasson^59^	2014	RCT	341	10	Other, colon, pancreatic	First‐line chemotherapy (>80%)	Mini Nutritional Assessment score <17	Dietary counselling	SOC	Mortality	Food records
Pottel^60^	2014	RCT	91	8	H&N	Radiotherapy	WL > 5% or >2% and BMI < 20 kg/m^2^	Echium oil 7.5 mL b.i.d. during treatment (omega‐3)	Placebo (oil same amount without omega‐3)	BW	EORTC QLQ C‐30 (appetite item)
Poulsen^61^	2014	Open‐label RCT	61	5	Gastric, oesophageal, gynaecological	No, chemotherapy and/or radiotherapy in palliative or curative intent	NA	Dietary counselling + protein supplement with EPA	SOC	Not defined	EORTC QLQ C‐30 (appetite item)
Focan^62^	2015	RCT	53	7	Breast, other, GI	NR	WL 2% 1 month or 5% 6 months, BMI < 20, low Alb/pre‐Alb, elevated CRP	Dietetic and psychological mindfulness workshops	SOC	Not defined	EORTC QLQ C‐30 (appetite item)
Gavazzi^63^	2016	Open‐label RCT	79	7	GI (56% gastric)	Planned surgery, additional chemotherapy in 30 patients	NRS2002 ≥ 3	EN (night). Usual food (daytime)	Dietary advice and ONS. EN if WL ≥ 5% after 2 months	BW	Dietary history
Jatoi^64^	2016	RCT	141	8	Lung, GI, other	No, chemotherapy and/or radiotherapy	WL > 5 lb in 2 months or intake <20 kcal/kg BW/day	White wine twice a day	Nutritional supplement	Appetite	NCCTG (appetite questions)
Takayama^65^	2016	Double‐blind RCT	181	8	NSCLC	(Not high‐emetic risk) palliative chemotherapy	WL ≥ 5% in 6 months; two of the following: Anorexia, fatigue, malaise, decreased muscle strength, arm muscle circumference <10th percentile. One of the following: CRP > 5.0 mg/L, Hb < 12 g/dL or Alb < 3.2 g/dL	(1) Anamorelin 50 mg (2) Anamorelin 100 mg	(3) Placebo	LBM and grip strength	Appetite QoL‐ACD
Woo^66^	2016	RCT	67	9	Pancreatic	Chemotherapy, radiotherapy or no treatment.	WL 5–10% in the last 3 months	Pancreatic exocrine replacement therapy	Placebo	BW	EORTC QLQ C‐30 (appetite item), food records
Jatoi^67^	2017	Double‐blind RCT	263	8	Other, lung, GI	With/without chemotherapy	WL > 5 lb in 2 months or intake <20 kcal/kg BW/day	Creatine	Placebo	BW	NCCTG (appetite questions)
Kapoor ^68^	2017	Open‐label RCT	63	8	Female genitourinary, other, breast	Not described	Advanced cancer, WL > 5% pretreatment weight, BMI < 20, Hb < 12 g/dL, energy intake <1500 kcal/day Dietary advice	Dietary counselling + Improved Atta (IAtta) (flour mix)	Dietary advice	BW, body composition and QoL	EORTC QLQ C‐30 (appetite item), 24‐h recall
Leedo^69^	2017	Open‐label RCT	40	8	Lung	Chemotherapy and/or radiotherapy or no treatment	NRS2002 ≥ 3, life expectancy >12 weeks	Dietary counselling	SOC	QoL	Dietary history
Werner^70^	2017	Double‐blind RCT	60	7	Pancreatic	In both groups, 12 received chemotherapy and one was treated with radiation	WL > 5% since diagnosis	Capsules: Marine omega‐3 fatty acids as phospholipids; 6.9% EPA and 13.6% DHA	Capsules: Marine omega‐3 fatty acids as triglycerides; 6.9% EPA and 13.6% DHA	BW and appetite	EORTC QLQ C‐30 (appetite item)
Zietarska^71^	2017	RCT	95	6	Colorectal	Chemotherapy	Asymptomatic pre‐cachexia according to SCRINIO Working Group	ONS	SOC	Chemotherapy‐related toxicity	Appetite VAS
Katakami^72^	2018	Double‐blind RCT	174	8	NSCLC	No, chemotherapy/TKI therapy and/or radiotherapy	WL ≥ 5% in 6 months, anorexia and two of the following: fatigue, muscle weakness, arm muscle circumference <10th percentile And one of the following: Alb < 3.2 g/dL, CRP > 5 mg/L, Hb < 12 g/dL	Anamorelin	Placebo	LBM	Appetite QoL‐ACD
Kouchaki^73^	2018	Double‐blind RCT	90	8	GI (52% gastric)	No, prior surgery and/or concomitant chemotherapy	WL ≥ 5% past 6 months or premorbid stable weight or BMI < 20 kg/m^2^	MA + celecoxib	MA + placebo	BW	Appetite VAS, EORTC QLQ C‐30 (appetite item)
Schink^74^	2018	Non‐randomized	131	9	Other, colon, lung	Systemic therapy, radiotherapy or combinations	NA	Exercise + dietary counselling	Nutritional advice only	Skeletal muscle mass	EORTC QLQ C‐30 (appetite item)
Turcott^75^	2018	Double‐blind RCT	47	7	NSCLC	Chemotherapy	FAACT ACS	Nabilone	Placebo	FAACT ACS	Appetite VAS, EORTC QLQ C‐30 (appetite item)
Uster^76^	2018	RCT	58	9	Lung, GI, other	Not described	NA	Dietary counselling + exercise	SOC	QoL	EORTC QLQ C‐30 (appetite item), food records
Britton^77^	2019	Cluster RCT	307	8	H&N	(Chemo)radiotherapy, primary or (neo)adjuvant	NA	Motivational interview and cognitive behavioural therapy	SOC	PG‐SGA	EORTC QLQ C‐30 (appetite item)
Cereda^78^	2019	RCT	166	8	Lung, pancreatic, other	First‐line chemotherapy (>90%)	NA	Dietary counselling with whey protein isolate	Nutritional counselling	Phase angle	24‐h recall, food records
Laviano^79^	2019	Double‐blind RCT	55	8	NSCLC	Chemotherapy	WL 2.5–11% in the last 12 months	*n*‐3 PUFAs (2 g), 25‐hydroxy vitamin D_3_ and high‐quality whey protein	Isocaloric drink based on milk and sunflower oil	Safety and tolerability	Appetite NRS (Council on Nutrition and Appetite)
Obling^80^	2019	RCT	47	7	GI (58% pancreatic)	Palliative chemotherapy (>90%)	NRS 2002 ≥ 2	Supplemental home PN	Dietary advice to reach 75% of estimated energy and protein needs	Fat‐free mass	24‐h recall
Bouleuc^81^	2020	RCT	148	7	Other, GI, lung	Ongoing chemotherapy 45%	Median WL 8.2 kg in the previous 6 months, mean food intake 40%	PN	ONS	QLQ C15 PAL (overall QoL, physical function and fatigue)	EORTC QLQ C15 PAL (appetite item)
Dehghani^82^	2020	Single‐blind RCT	43	7	Gastric	All treated with chemotherapy	BMI < 18 kg/m^2^ and muscle wasting	Captopril	Placebo	QLQ C30, not specified item/scale	EORTC QLQ C‐30 (appetite item)
Famil‐Dardashti^83^	2020	Double‐blind RCT	47	8	GI, lung, other	NA	WL ≥ 5% within 2 months, ≥3 months of life expectancy	Herbal combination (fenugreek, fennel, chicory) + MA	Placebo + MA	BW	Appetite NRS (ESAS)
Hong^84^	2020	Quasi‐RCT	204	9	GI (27% colon)	Chemotherapy or concomitant radiotherapy	NA	Resistance exercise	Relaxation exercise	Physical function	EORTC QLQ C‐30 (appetite item)
Movahed^85^	2020	Open‐label RCT	100	8	Oesophageal	Chemoradiation	Newly diagnosed oesophageal cancer, candidate for radiotherapy or chemoradiation	Individualized nutritional plan based on needs ONS when needs not met	General dietary advice	PG‐SGA score	24‐h recall
Qiu^86^	2020	RCT	96	6	Oesophageal	NA	NA	Whole‐course nutrition management	SOC	Not defined	EORTC QLQ C‐30 (appetite item)
Storck^87^	2020	RCT	52	10	Lung, other, pancreatic	Palliative breast and prostate cancer received chemotherapy	NA	Physical exercise (3 × week endurance + strength), whey protein supplement	SOC	Short Physical Performance Battery	EORTC QLQ C‐30 (appetite item), food records
Bargetzi^88^	2021	Open‐label RCT	506	8	Other, lung, haematological	Ongoing cancer treatment (>86%)	NRS 2002 ≥ 3	Early individual nutritional support	SOC	Mortality	Food records
Currow^89^	2021	Double‐blind RCT	190	6	Lung, other, colorectal	NA	Appetite ≤4 NRS	(1) MA (2) Dexamethasone	(3) Placebo	Appetite NRS	Appetite NRS (MSAS)
Hunter^90^	2021	Double‐blind RCT	120	7	Other, pancreatic, breast	With and without chemotherapy	WL > 5% over 6 months or >2% over 6 months and BMI < 20 kg/m^2^	Mirtazapine	Placebo	Appetite	Appetite NRS
Izumi^91^	2021	RCT	81	6	Renal, other	Systemic therapy, radiotherapy, surgery	NA	Testosterone	SOC		Appetite NRS (ESAS)
Ko^92^	2021	RCT	40	8	NA	>1 month after chemotherapy	Appetite VAS ≥ 4	Yukgunja‐Tang + dietary counselling	Nutritional counselling	FAACT ACS	Appetite VAS
Molassiotis^93^	2021	Pilot RCT Two trials	74	9	GI, other, urological	Not described	Stage III or IV, ECOG ≤ 2, at risk of malnutrition (MST)	Family‐centred nutritional intervention with a dietitian	SOC	PG‐SGA (SF), energy and protein intake, FAACT and QoL	Food records
Kutz^94^	2022	RCT	69	7	H&N	(Chemo)radiotherapy, primary or adjuvant	NA	Dietary counselling	SOC	Phase angle	EORTC QLQ C‐30 (appetite item)

Abbreviations: Alb, albumin; ATP, adenosine‐5′‐triphosphate; b.i.d., bis in die (twice a day); BMI, body mass index; BSC, best supportive care; BW, body weight; CBD, cannabidiol; COX, cyclooxygenase; CRP, C‐reactive protein; DHA, docosahexaenoic acid; ECOG, Eastern Cooperative Oncology Group; EN, enteral nutrition; EORTC QLQ, European Organisation for Research and Treatment of Cancer Quality of Life Questionnaire; EPA, eicosapentaenoic acid; EPO, erythropoietin; ESAS, Edmonton Symptom Assessment System; FAACT ACS, Functional Assessment of Anorexia/Cachexia Therapy Anorexia/Cachexia Subscale; GI, gastrointestinal; H&N, head and neck; Hb, haemoglobin; HCP, healthcare personnel; LASA, Linear Analogue Self‐Assessment; LBM, lean body mass; MA, megestrol acetate; MDASI, MD Anderson Symptom Inventory; MPA, medroxyprogesterone acetate; MSAS, Memorial Symptom Assessment Scale; MST, Malnutrition Screening Tool; NA, not applicable; NCCTG, North Central Cancer Treatment Group; NRS 2002, Nutrition Risk Screening 2002; NRS, numerical rating scale; NSCLC, non‐small cell lung cancer; ONS, oral nutritional supplement; OS, overall survival; PG‐SGA (SF), Patient‐Generated Subjective Global Assessment (Short Form); PN, parenteral nutrition; PUFA, polyunsaturated fatty acid; QoL, quality of life; QoL‐ACD, Quality of Life Questionnaire for Cancer Patients Treated with Anticancer Drugs; RCT, randomized controlled trial; REE, resting energy expenditure; SOC, standard of care; SSA, subjective sense of appetite; t.i.d., ter in die (three times a day); THC, tetrahydrocannabinol; TKI, tyrosine kinase inhibitor; VAS, visual analogue scale; WHO PS, World Health Organization Performance Status; WL, weight loss.

^a^ Number randomized.

Trials had an intervention period lasting from 2 weeks to 2 years. Of the eligible trials, 65 (81%) measured appetite endpoints, and 26 (40%) showed statistically significant improvements. In 15 studies (23%), appetite was a primary or co‐primary endpoint, whereof 6 (40%) presented statistically significant results in favour of the intervention. Dietary intake endpoints were assessed in 21 (26%) trials, and 12 (57%) showed statistically significant improvements. Seven (33%) had dietary intake as the primary or co‐primary endpoint(s), whereof 4 (57%) were statistically significant in favour of the intervention. Body weight was the most common co‐primary outcome alongside appetite or dietary intake. Sample size calculations were conducted in 12 (54%) of the 22 trials, which had appetite or dietary intake as a primary endpoint (11 [73%] on appetite and 1 [14%] on dietary intake). Six (8%) trials used both appetite and dietary intake endpoints. Five (8%) and 2 (10%) trials used two methods to assess appetite and dietary intake, respectively.

**Figure 2:**
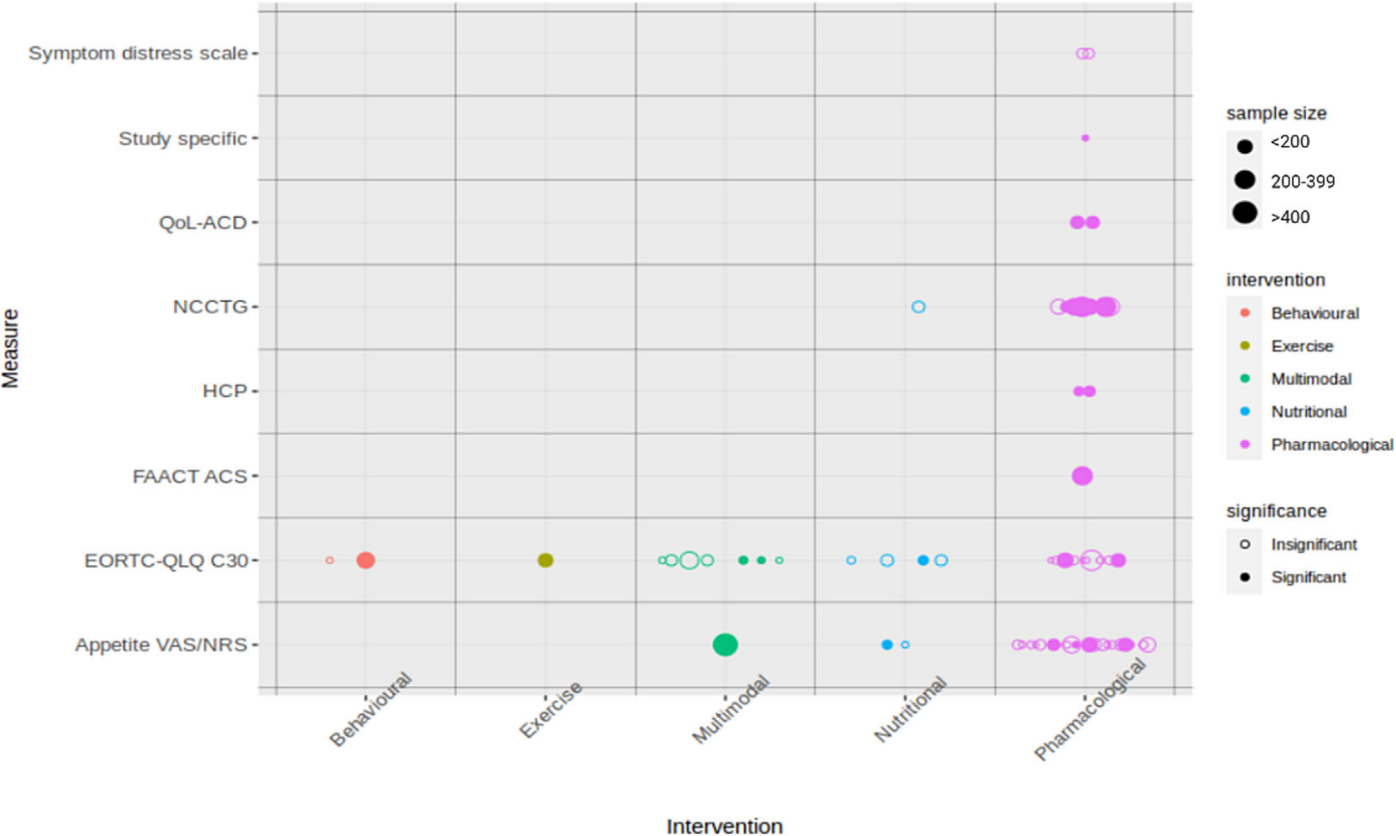
The relationship between study interventions, sample size, statistical significance and appetite measures across eligible trials. EORTC‐QLQ C30, European Organisation for Research and Treatment of Cancer Quality of Life Questionnaire C30/C15 PAL; FAACT A/CS, Functional Assessment of Anorexia/Cachexia Therapy Anorexia/Cachexia Subscale; HCP, healthcare personnel score; NCCTG, North Central Cancer Treatment Group; QoL‐ACD, Quality of Life Questionnaire for Cancer Patients Treated with Anticancer Drugs; VAS/NRS, visual analogue scale/numerical rating scale.

**Figure 3:**
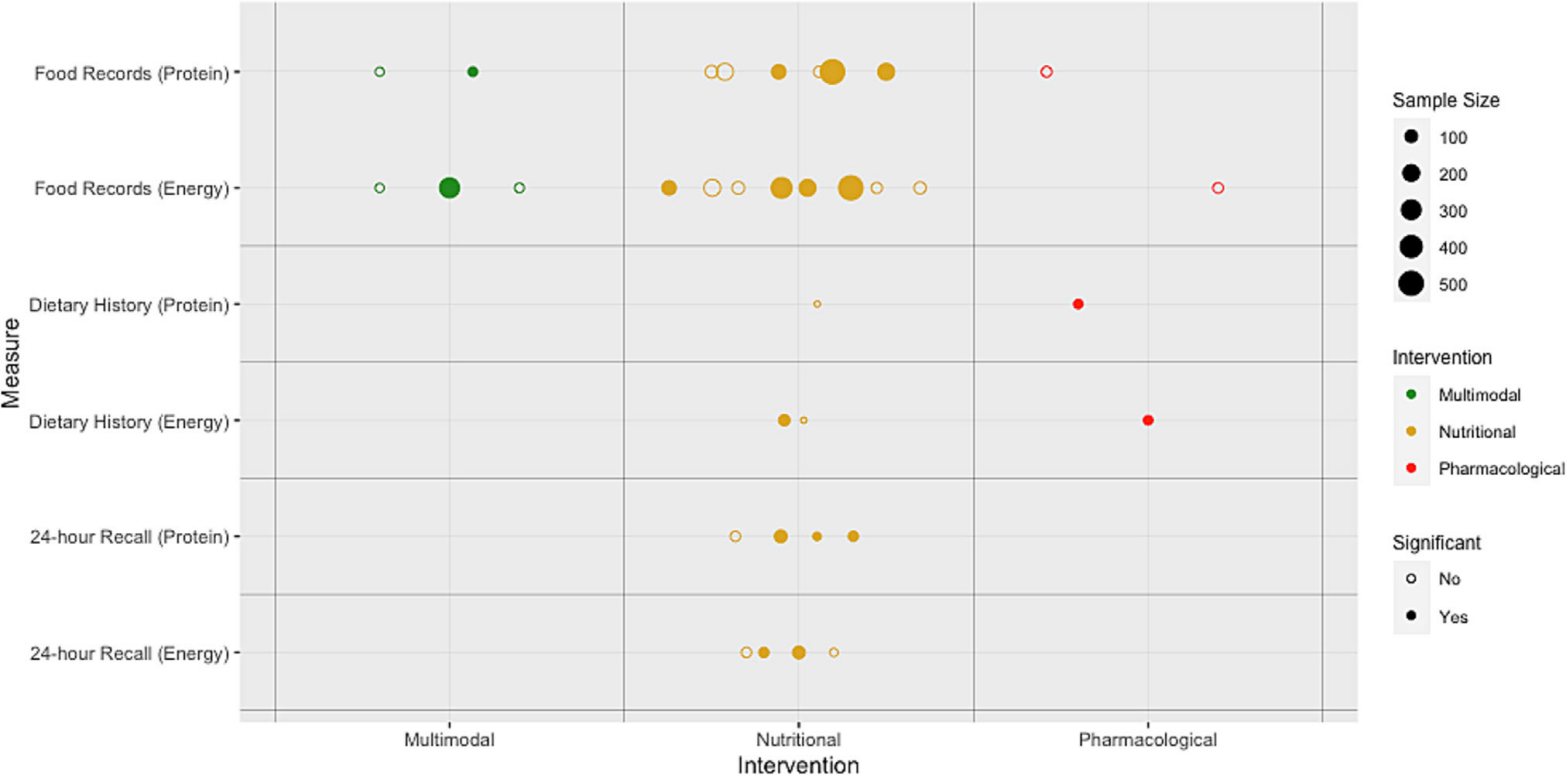
The relationship between study interventions, sample size, statistical significance and dietary intake measures across eligible trials.

Appetite was primarily investigated in pharmacological interventions (41 studies), while dietary intake was mainly assessed in nutritional intervention trials (14 studies). *Figures* *2* and *3* illustrate the relationship between the type of study intervention utilized, sample size, and the presence of significant findings related to appetite or dietary intake endpoints.

### Appetite endpoints

*Table* *2* details the frequency of appetite and dietary intake endpoints used in eligible trials. The most frequent endpoint for assessment of appetite was visual analogue scale (VAS) or numerical rating scale (NRS) (26/65 [40%]). The appetite questions from the EORTC QLQ C30/C15 PAL (25 studies [38%]), as well as the appetite question from North Central Cancer Treatment Group (NCCTG) anorexia questionnaire (11 studies [17%]), were often applied.

**Table 2:** Frequency of use of appetite and dietary intake endpoints in eligible trials

Endpoint	Description and time frame	*N* (trials)	Year	Total sample Median (min–max)	Type of intervention	Trials with significant results for appetite or dietary intake endpoints			References	
						*N* (trials)	Total sample (min–max)	Type of intervention	Sign	Ns
Appetite endpoints										
VAS/NRS	VAS (0–100 mm) and NRS (0–10) Appetite now–last week–lately–these days	26	1992–2021	3510 77 (40–628)	Pharmacological: 22 Nutritional: 3 Multimodal: 1	8	1468 (42–628)	Pharmacological: 6 Nutritional: 1 Multimodal: 1	^17^, ^18^, ^25^, ^26^, ^28^, ^57^, ^71^, ^83^	^27^, ^31^, ^37^, ^45^, ^49^, ^50^, ^53^, ^54^, ^55^, ^56^, ^58^, ^73^, ^75^, ^79^, ^89^, ^90^, ^91^, ^92^
EORTC QLQ C30/C15 PAL (appetite item)	During the past week: ‘Have you lacked appetite?’ 0–4 Likert scale ‘not at all’ to ‘very much’	25	1996–2022	3396 93 (43–518)	Pharmacological: 7 Exercise: 1 Nutritional: 8 Multimodal: 7 Behavioural: 2	8	1267 (61–307)	Pharmacological: 2 Exercise: 1 Nutritional: 2 Multimodal: 2 Behavioural: 1	^25^, ^32^, ^43^, ^61^, ^68^, ^77^, ^84^, ^86^	^31^, ^35^, ^44^, ^51^, ^52^, ^60^, ^62^, ^66^, ^70^, ^73^, ^74^, ^75^, ^76^, ^81^, ^82^, ^87^, ^94^
NCCTG (appetite questions)	Appetite now compared to before illness (‘increased’ to ‘markedly reduced’) Appetite now compared to before study medication (‘reduced’ to ‘increased very much’) How would you rate/describe your appetite? ‘very good’ to ‘very poor’	11	1990–2017	2752 263 (61–496)	Pharmacological: 9 Nutritional: 2	5	1733 (133–496)	Pharmacological: 5	^15^, ^16^, ^19^, ^30^, ^34^	^22^, ^39^, ^46^, ^48^, ^64^, ^67^
QoL‐ACD (Item 8)	In the past few days: ‘Did you have good appetite?’ 1–5 (‘not at all’ to ‘very much’)	2	2016–2018	354 178 (174–181)	Pharmacological: 2	2	354 (174–181)	Pharmacological: 2	^65^, ^72^	‐
Symptom Distress Scale (Item 3)	Appetite lately 1–5 Likert statements from ‘I have my normal appetite and enjoy good food’ to ‘I cannot stand the thought of food’	2	1996–2002	241 (119–122)	Pharmacological: 2	‐	‐	‐	‐	^24^, ^36^
Scored by HCP	Scored 1–5 based on interview	2	1996–2000	229 (100–129)	Pharmacological: 2	2	229 (100–129)	Pharmacological: 2	^23^, ^33^	‐
FAACT (Item C6)	In the past 7 days: ‘I have good appetite’ 0–4 (‘not at all’ to ‘very much’)	1	2004	421	Nutritional: 1	1	421	Nutritional: 1	^39^	‐
Study specific	Appetite 1–5 ‘none’, ‘poor’, ‘fair’, ‘good’, ‘excellent’	1	1994	52	Pharmacological: 1	1	52	Pharmacological: 1	^21^	‐
Dietary intake endpoints										
Food records	Prospective recording (usually 3–4 days) of all foods, beverages and dietary supplements consumed within a designated period	13	1993–2021	2269 183 (52–506)	Pharmacological: 2 Nutritional: 8 Multimodal: 3	Energy: 5	1493 (105–506)	Nutritional: 4 Multimodal: 1	Energy: ^20^, ^40^, ^41^, ^59^, ^88^	^37^, ^38^, ^47^, ^66^, ^78^, ^87^, ^93^
						Protein: 4	901 (58–506)	Nutritional: 3 Multimodal: 1	Protein: ^20^, ^41^, ^76^, ^88^	
24‐h recall	Retrospective assessment of intake in the previous 24 h	6	2005–2020	515 64 (47–166)	Nutritional: 6	Energy: 3	238 (63–100)	Nutritional: 3	Energy: ^43^, ^68^, ^85^	^42^, ^78^
						Protein: 4	285 (47–100)	Nutritional: 4	Protein: ^43^, ^68^, ^80^, ^85^	
Dietary history	Retrospective interview about usual intake of foods and drinks over a specified period	4	1998–2017	248 65 (40–79)	Nutritional: 3 Pharmacological: 1	Energy: 2	133 (54–79)	Pharmacological: 1 Nutritional: 1	Energy: ^29^, ^63^	^43^, ^69^
						Protein: 1	54	Pharmacological: 1	Protein: ^29^	

Abbreviations: EORTC QLQ, European Organisation for Research and Treatment of Cancer Quality of Life Questionnaire; FAACT, Functional Assessment of Anorexia/Cachexia Therapy; HCP, healthcare personnel; NCCTG, North Central Cancer Treatment Group; NRS, numerical rating scale; Ns, non‐significant; QoL‐ACD, Quality of Life Questionnaire for Cancer Patients Treated with Anticancer Drugs; Sign, statistically significant; VAS, visual analogue scale.

The most frequent intervention was progestin treatment with either megestrol acetate (MA) or medroxyprogesterone acetate (MPA) (10 studies with a placebo control arm). These studies measured appetite with four different assessment tools, and comparison of effect sizes to assess whether one of these tools was more sensitive to changes was not possible.

#### Visual analogue scale and numerical rating scale

VAS and NRS were categorized together in this review as it was challenging to interpret which of the two had been applied. Twenty‐two of the 26 studies were pharmacological, as were six of the eight studies presenting statistically significant results. VAS/NRS was the primary endpoint in 9 of the 26 studies, and 3 of these studies reported statistically significant results (*Table* *2*). Twenty of the 26 studies also examined body weight. In 14 of these trials, results for body weight and appetite were aligned in that both were either statistically significantly different in favour of the intervention or statistically non‐significant. In the remaining six studies, there were statistically significant differences in only one of the endpoints (appetite [three studies] or weight [three studies]). Only 1 of the 26 studies reported both dietary intake and appetite, and results were statistically non‐significant for both endpoints.

#### European Organisation for Research and Treatment of Cancer Quality of Life Questionnaire C30/C15 PAL

The use of EORTC QLQ C30/C15 PAL was more equally distributed between trials exploring a wide variety of different interventions (*Figure* *2*). Eight of the 25 studies assessing appetite using EORTC QLQ C30/C15 PAL showed statistically significant differences in appetite between the study arms in favour of the intervention. In two studies, appetite was defined as the primary outcome, and one of these studies reported a statistically significant improvement in appetite.

Sixteen of the 25 studies measuring appetite with the EORTC QLQ C30/C15 PAL also reported results for weight; 11 studies reported either changes between arms in both endpoints and in the same direction or no change in both endpoints. Five studies described statistically significant changes between the arms, in either appetite or weight, but not for both endpoints. Five of the 25 studies assessed both dietary intake and appetite. In one study, there were significant changes between the arms for both endpoints, and in three studies, there were non‐significant differences. In one study, appetite was non‐significant, while dietary intake was significant.

#### North Central Cancer Treatment Group anorexia questionnaire

NCCTG anorexia questionnaire was used to assess appetite in large, predominantly pharmacological studies. Some of the studies used slightly modified versions of the questionnaire. Five of the 11 studies presented statistically significant differences between the treatment arms. Three studies used NCCTG anorexia questionnaire as primary outcome measure for appetite, whereof two studies presented statistically significant results.

### Dietary intake endpoints

As presented in *Table* *2*, food records were the method most often used to assess dietary intake (13 out of 21 trials [62%]). Six trials (29%) used 24‐h recall, and four trials (19%) used dietary history method. One trial combined food record with 24‐h recall, and one trial combined 24‐h recall with dietary history.

Fifteen of the 21 trials (71%) investigated effects of various nutritional interventions. Three pharmacological intervention trials and three multimodal intervention trials assessed dietary intake to determine whether improved appetite or other symptoms affected dietary intake.

#### Food records

Of the 13 trials using food records, eight trials were nutritional interventions assessing energy and/or protein intake mainly to evaluate compliance with food‐based advice (*Table* *2*). Four (50%) of these eight trials reported a statistically significantly higher energy intake in the intervention group than in the control group, while three trials reported statistically significantly higher protein intake. In two trials the comparators were standard of care, in one trial isocaloric oral nutritional supplements (ONS) without *n*‐3 polyunsaturated fatty acids (PUFAs) and in the last one the comparator was a daily multivitamin‐mineral tablet. Of the remaining five trials using food records (three multimodal and two pharmacological), one multimodal trial (parenteral nutrition [PN], cyclooxygenase [COX] and erythropoietin [EPO]) showed statistically significantly higher energy intake in the intervention group. In the four trials showing no statistically significant difference in energy and protein intake between arms, two used isocaloric comparators (ONS with or without *n*‐3 PUFAs and capsules with or without *n*‐3 PUFAs). The other two trials presented no statistically significant effect of either family‐centred psychosocial nutritional intervention or multimodal intervention (exercise and whey protein supplement) compared to standard of care.

Twelve of the 13 trials assessed dietary intake and body weight. Seven trials consistently showed a pattern where dietary intake corresponded to changes in body weight, and in two of these trials, both energy and protein intake increased significantly and corresponded to body weight gain, favouring the treatment arm. In the remaining five trials, no significant changes were observed in either dietary intake or body weight between the study arms. However, in the other five trials, a diversity pattern was observed. In one trial, a significant increase in energy intake coincided with a decline in body weight, while in two other trials, it corresponded to no change in body weight. Moreover, significantly increased protein intake did not correspond to changes in body weight. Finally, one study that used both food records and 24‐h dietary recall reported no increase in energy or protein intake, while body weight increased. Four trials assessed both dietary intake and appetite. In one trial, there was a statistically significant improvement in protein intake but no change in appetite. The three remaining studies showed no changes in either endpoint.

#### 24‐h dietary recall

Six nutritional trials used 24‐h recall to assess intake. Three trials that compared dietary counselling with or without ONS showed higher energy and protein intake in favour of the intervention. The comparators in these trials were standard of care or dietary advice. Further, in one trial, a higher protein intake was shown by supplemental PN compared to dietary advice. In the remaining two trials, no differences in energy or protein intake were found, and comparators were dietary counselling, enriched oral diet or enteral nutrition (EN).

Five trials using 24‐h recall assessed dietary intake and body weight. In two trials, both energy and protein and body weight increased significantly. Among the remaining three trials, one reported a significant increase in energy and protein intake but did not observe a significant change in body weight. Further, one trial showed no change in dietary intake or body weight. One trial using both 24‐h recall and food records showed no significant increase in energy or protein intake, although body weight increased. Two trials assessed both dietary intake and appetite, and a significant increase in energy and protein intake, as well as in appetite, was observed in both. Additionally, this trial also reported a significant increase in body weight.

#### Dietary history

Four trials used retrospective interviews to assess the usual consumption of foods and drinks over a specified period. In one pharmacological trial, significantly higher energy and protein intake in favour of the intervention were reported, and in one EN trial, significantly higher energy intake was reported. Another trial reported significantly higher energy and protein when using 24‐h recall, but not the dietary history method.

In three trials, body weight was also reported. In the trial using EN, the significant increase in energy intake corresponded to a significant increase in body weight. Additionally, one trial reported no increase in energy or protein intake or body weight. The trial using both dietary history and 24‐h recall showed no statistically significant increase in energy or protein intake, although body weight increased. None of the trials assessing dietary history also assessed appetite.

### Effect size/sample size estimation

Available raw scores (baseline, post‐treatment and changes) for all studies are shown in *Tables* *S1*–*S4*. Eight studies on appetite (4 VAS/NRS, 3 EORTC and 1 QoL‐ACD) reported sufficient data (mean change and SD) to calculate effect size (Hedge's *g*) (*Tables* *S1* and *S2*). Assuming that Hedge's *g* of 0.2, 0.5 and 0.8 corresponds to a small, medium or large effect size, one can use the pooled standard deviations for the control groups for VAS/NRS and EORTC to estimate the corresponding changes on these two scales (estimated needed sample size per arm for 80% power and alpha of 0.05 in parentheses): For VAS/NRS (0–10), a mean change in score of 0.5 (*n* = 362), 1.3 (*n* = 58) and 2.2 (*n* = 23) corresponds to a small, medium or large effect size, respectively. For EORTC (0–100), a mean change in score of 7 (*n* = 331), 18 (*n* = 53) and 29 (*n* = 21) corresponds to a small, medium or large effect size, respectively.

Five studies assessing energy intake (four with food records and one with 24‐h recall) and four studies assessing protein intake (three with food records and one with 24‐h recall) reported sufficient data to calculate effect sizes (*Tables* *S3* and *S4*). Based on three studies using the same unit of kcal/day assessed with food records, a mean change of 93 kcal/day (*n* = 427), 231 kcal/day (*n* = 68) and 370 kcal/day (*n* = 27) represents a small, medium and large effect size, respectively. Based on the same three studies reporting protein intake in g/day, a mean change of 5 g/day (*n* = 370), 11 g/day (*n* = 59) and 18 g/day (*n* = 23) represents a small, medium and large effect size, respectively.

## Discussion

This review identified a multitude of measures that have been used to assess nutritional aspects in cancer cachexia clinical trials. VAS/NRS and the EORTC QLQ C30/C15 PAL were the most commonly used endpoints for assessment of appetite, but measures of appetite were rarely the primary endpoint (23%). About 40% of studies reported a statistically significant improvement in appetite in favour of the intervention. In 70% of the studies, changes in appetite and weight coincided. Dietary intake tended to be used more for monitoring compliance with dietary interventions than as a primary endpoint per se. Prospective food records were the most frequently used method to assess dietary intake of energy and/or protein. Statistically significantly higher energy and/or protein intakes in favour of the intervention group were reported in ~60% of trials, and increased dietary intake coincided with weight gain or stabilization in approximately half of these. Very few studies reported both dietary intake and appetite.

Appetite is generally defined as a person's desire to eat and is a subjective feeling that is best assessed with a PROM,^95^ which was the case in nearly all trials included in this review. Two main groups of PROMS were identified: VAS/NRS and categorical response scales (CRSs), such as EORTC QLQ C30/C15 PAL and NCCTG anorexia questionnaire. VAS/NRS asks the respondent to grade their appetite loss somewhere between two extremities (no appetite loss vs. worst appetite loss), while CRS asks the respondent to pick the most fitting response out of a set of (usually) four or five available responses that are ordered in terms of intensity of appetite loss. For the purpose of this review, VAS and NRS results were grouped together. Many prior studies have shown a high degree of correlation between VAS and NRS,^96^ and it is not clear which is optimal. When assessing appetite, NRS is a well‐known, user friendly and validated assessment, and both its prognostic abilities and minimally clinically important difference have been evaluated.^96^ A relatively small study from a palliative care unit reported that VAS was the least preferred appetite scale by patients, while CRS was favoured, slightly more than NRS.^97^ NRS or EORTC could thus preferably be recommended when assessing appetite in clinical trials.

In a bid to assess the multifactorial aspects of appetite loss, some studies used multidimensional assessment tools such as the FAACT questionnaire. However, studies often only reported the summary score, which is influenced by several factors, making it difficult to assess the effect on appetite per se and therefore were not included in the present review Of note, the FAACT questionnaire has been refined to a five‐item anorexia symptoms subscale (5‐IASS), and this is being assessed in ongoing clinical trials of anamorelin, mirtazapine and MA.^12^ The EORTC QLQ CAX24, assessing aspects of appetite and QoL in patients with cachexia, has been validated, and a report is awaited (https://qol.eortc.org/questionnaire/qlq‐cax24/); no studies in this review used this tool. Whether 5‐IASS or EORTC QLQ CAX24 will better capture treatment effect is so far unclear. By all accounts, scoring of patients' symptoms by healthcare professionals, like in two studies in this review, should be discouraged.

There is no agreement on how to best measure food intake in patients with cancer,^98^ and currently, assessments of dietary intake have not been validated in the cachexia population. Extrapolation from validations in healthy populations is not adequate because dietary intake can vary during the disease and the anti‐cancer treatment trajectory, and there is no agreement when assessment should be performed. Further, due to the catabolic phenotype and muscle wasting in cachexia, sensitive and specific methods validated for estimation of energy and protein intake are needed for trials in this specific patient population.

Weight remains a clinical and relevant measure of energy balance; however, the required energy and protein needed in cancer cachexia to maintain or increase body weight remain unknown. In the current review, only half of the trials showed that intake and body weight changed in the same direction, and when two different methods were applied in the same trial, inconsistencies in results were reported. This highlights a variability in the agreement between dietary intake methods and body weight. Another important aspect of dietary intake methods is that we lack prognostic and predictive value of reduced intake, including relevant prognostic thresholds. This limits dietary intake from serving as a clinically meaningful primary endpoint in cancer cachexia trials, as a statistically significant increase in energy and protein alone may not reflect clinically relevant changes due to the underlying catabolic pathophysiology. Nevertheless, dietary intake assessment is important to monitor and guide nutritional intervention as well as compliance. The superiority of any dietary intake method cannot be concluded based on this review. Additionally, simplified assessments that did not provide detailed reports on calorie and protein intake were not included in this review, as they cannot quantify individual calorie or protein intake in kcal/day or g/day. More simplified methods could be considered when appetite and/or body weight are the main endpoints, but also, these need to be validated with concern to energy and protein intake in the cancer cachexia population.

One of the challenges in assessing appetite and dietary intake in cachexia is the complex interplay between these and confounding factors. Nutritional impact symptoms such as altered sense of smell or taste, early satiety and nausea/vomiting may undermine the desire to eat, yet these were rarely assessed in the reviewed trials. Furthermore, energy expenditure and/or unmitigated catabolism relative to dietary intake of energy/protein determines whether weight can be gained. All these factors are important and need to be collectively understood. This seemingly motivates the inclusion of multiple endpoints in studies of cachexia, which was the case for many of the studies in this review. However, well‐defined primary endpoints are essential, and inclusion of secondary endpoints should be balanced against the risk of spurious findings and preferably be restricted to endpoints of relevance to understand changes in the primary endpoint.

The primary endpoint for about half of the studies in this review was body weight or composition. This is not surprising as weight loss and body composition are key factors defining cachexia.^1^ Appetite or dietary intake was only used as primary endpoint in ~25% of trials, including studies using appetite and dietary intake as co‐primary outcomes with weight/body composition. However, although the 2011 international definition on cancer cachexia emphasized that nutritional intervention alone cannot fully reverse cachexia,^99^ stakeholders recognized the importance of dietary intake and appetite improvement as part of any cachexia management.^100^ Appetite and dietary intake could thus be considered important co‐primary or secondary endpoints in the context of cachexia treatment.

Contrary to body weight and composition, appetite and dietary intake are relevant endpoints throughout the entire cancer trajectory. Interventions against cachexia late in the disease trajectory will have low probability of improving muscle mass or weight due to minimal anabolic potential in patients with a short remaining life expectancy.^0099^ In contrast, improving appetite and increasing dietary intake is shown to improve QoL and reduce emotional distress among patients and relatives alike, through the whole disease trajectory.^100^ Consequently, appetite and dietary intake are key in each phase of the disease, and intervention trials aiming to improve either of the two should measure appetite and/or dietary intake. Albeit, at the very end of life, preservation of appetite probably becomes more important than amount of energy and protein intake, as the participation in meals and enjoyment of food still are of great value.

When selecting endpoints for a study, the intervention's mechanism of action and the comparators should be considered. For instance, if the effect of an appetite stimulant is evaluated, body weight should not be the only endpoint, as poor appetite alone rarely is the only cause of weight loss in patients with cancer.^100^ In such a case, it would be natural to choose appetite as the primary endpoint and weight or dietary intake as a secondary or exploratory endpoint. Dietary intake can serve multiple purposes depending on the specific goals of the trial and the research questions being addressed. Because of lack of validation in cachexia populations, dietary intake has limited value as primary efficacy endpoint. However, in exploratory studies, measurements of energy intake can be used to better understand energy balance, helping researchers addressing cancer cachexia pathophysiology beyond trial endpoints. Dietary intake also could have value as a measure of patient compliance.

The relationship between dietary intake and appetite has been sparsely investigated in patients with cancer, and in the present review, only 8% of the trials measured both, and there may be several reasons for this. Dietary intake is often more time and resource demanding to assess than appetite, and it is sometimes assumed that appetite and dietary intake are two sides to the same story. However, it is important to recognize that although appetite and intake are correlated, some manage to keep a stable dietary intake despite loss of appetite,^101^ and thus, endpoints of appetite and dietary intake cannot be used interchangeably or as substitutes for one another. This is also seen in the present review where in studies where weight and dietary intake were assessed together, direction of change in both endpoints coincided in only half of the studies, indicating no real association between the endpoints used to evaluate these two outcomes. On the other hand, concordance between appetite and weight loss was more apparent as changes in weight and appetite coincided in 70% of studies. This could indicate that instruments used to measure appetite have reasonable construct validity; however, above all, this underscores the complexity of cachexia, indicating that dietary intake is not dependent on appetite alone, and weight is not solely dependent on appetite or dietary intake. It is not possible, based on data available, to state whether any of the different measurements of appetite or dietary intake corresponds closer to change in body weight than the others.

Equally important to choosing and prioritizing endpoints is to ensure an adequately sized study sample. Only half of the trials with appetite or dietary intake as primary endpoint reported sample size calculations. This may have led to inconclusive or underpowered trials that fail to provide reliable conclusions. In this review, we provide guidance to what might be appropriate sample sizes in relation to what is perceived to be small, moderate or large effect sizes. According to this, a sample size of <100, akin to approximately half of trials (37 of 80) in this review, is likely too small to detect moderate effect sizes concerning appetite or dietary intake.

The correct sample size depends on what is a clinically meaningful difference in the chosen endpoint. Approximately 40% of the trials reported statistically significant differences between the arms on appetite or dietary intake endpoints. Some of the studies, especially those where appetite and dietary intake were primary endpoints, discussed whether these differences also constituted a clinically important difference. However, there is no clear universal agreement on what a clinically important difference should be. Using VAS/NRS as an example, both a 15% difference between arms and a 25% increase from baseline have been used, as has a numerical difference of 1 and 3 on the 0–10 NRSs.^89^ Moderate to high effect sizes for energy intake as seen in this review  are sufficient for weight gain in healthy persons,^102^ but did not lead to increased body weight in the retrieved studies. Consequently, clinically meaningful changes in the cachexia population seem to be different from the healthy population, and are, as of yet, unknown.

### Strengths and limitations

The strengths of this review are its rigorous methodology and also the multinational and multiprofessional collaboration of experts behind it. This has ensured a wider range of inputs when evaluating the multidimensional condition that cancer cachexia is.

The heterogeneity in populations, interventions and usage of the different endpoints included in this review was substantial. Additionally, intervals from baseline to follow‐up assessments varied considerably between studies. Several studies did not describe whether they asked about appetite at worst or on average or which time frame the appetite question concerned (past month, last week or today).

The main inclusion criterion in most studies was unintentional weight loss, and only in relatively few of the analysed trials were loss of appetite and reduced dietary intake required. One could thus discuss if there is potential for improvement when there has been no deterioration in the endpoint prior to inclusion. However, the trials were still included as one could argue that an aim could be to prevent an expected deterioration in appetite and dietary intake in the intervention arm. Indeed, in populations where the prevalence and/or incidence of appetite loss and/or reduced intake is high, screening on these two parameters at study inclusion might not be necessary. However, in future studies aiming to treat appetite or improve dietary intake, this is something that needs to be considered closely.

Another limitation is that data in this review originate from clinical trials and not studies specifically designed to validate endpoints. Non‐significant statistical results do not imply that the trial's outcome measure was unsuitable but could mean that the study was underpowered, the design was wrong or the intervention had no impact on appetite or dietary intake. Also, the variations between interventions, their different targets and assessment methods made it impossible to determine whether any of the endpoints captured changes in appetite to a greater extent than others, also when exclusively comparing studies with the most frequently studied intervention MA/MPA. Nevertheless, when collating all studies published and exploring the endpoints in depth, the totality of studies can give a foundation to improve future study design.

## Conclusions

A variety of endpoints have been used to assess appetite and dietary intake in cancer cachexia clinical trials. The optimal nutritional endpoint cannot be determined based on the present review. VAS/NRS and the appetite item from EORTC QLQ C30/C15 PAL were the endpoints most frequently used to assess appetite, while food records were most used to assess dietary intake. From the trials assessed and available literature, NRS and EORTC QLQ C30/C15 PAL can be recommended as measures to assess appetite, either as a primary endpoint when the intervention primarily aims to target appetite or as a secondary measure where other factors that may influence appetite are being assessed. A next step for the academic community might be a formal process to establish an international consensus on which appetite endpoint(s) to use.

Few studies used energy and/or protein intake as a primary endpoint, and when used, it was mainly used to monitor interventions and compliance. This review and previous literature strongly suggest that appetite endpoints should not be used as a surrogate to assess dietary intake. Furthermore, dietary intake methods have not been validated in the present population, and the observed variability in the relationship between intake and body weight underscores the need for studies validating dietary intake in patients suffering from cancer cachexia. At present, dietary intake endpoints cannot be recommended as main efficacy endpoints in clinical trials evaluating treatment of cancer cachexia.

Several studies had multiple endpoints and a relatively small sample size, increasing the risk of spurious findings potentially entailing futile treatment or confirmatory studies with low probability of significant findings. Thus, future studies investigating appetite and dietary intake in patients suffering from, or at risk of developing cancer cachexia, should precisely define primary endpoint and perform sample size calculations.

## Conflict of interest statement

MF has received personal fees from Pfizer. MJH has received funding from CRUK, NIH National Cancer Institute, IASLC International Lung Cancer Foundation, Lung Cancer Research Foundation, Rosetrees Trust, UKI NETS and NIHR. MJH has consulted for, and is a member of, the Achilles Therapeutics Scientific Advisory Board and Steering Committee; has received speaker honoraria from Pfizer, Astex Pharmaceuticals, Oslo Cancer Cluster and Bristol Myers Squibb; and is a co‐inventor on a European patent application relating to methods to detect lung cancer (PCT/US2017/028013). RJES has received personal fees for consultancy from Artelo, Actimed, Faraday and Helsinn. BJAL has received personal fees for consultancy from Artelo, Actimed, Faraday, Kyowa Kirin and Toray. The remaining authors have not reported any conflicts of interest.

## Supporting information


**Appendix S1.** Documentation of literature search

**Appendix S2.** Modified Downs and Black Scoring Criteria

**Table S1.** Raw values of appetite scores pre‐ and posttreatment with delta, significance levels and effect sizes of two‐armed trials

**Table S2.** Raw values of appetite scores pre‐ and posttreatment with delta, significance levels and effect sizes of multi‐armed trials

**Table S3.** Raw values of dietary intake of energy and protein pre‐ and posttreatment with delta, significance levels and effect sizes of two‐armed trials

**Table S4.** Raw values of dietary intake of energy and protein pre‐ and posttreatment with delta, significance levels and effect sizes of multi‐armed trials
