# Supplementary material for: Appetite and dietary intake endpoints in cancer cachexia clinical trials: Systematic Review 2 of the cachexia endpoints series
Source: J Cachexia Sarcopenia Muscle. 2024 Feb 11;15(2):513–35. doi: 10.1002/jcsm.13434 (PMC10995275; doi:10.1002/jcsm.13434)
Supplement: Supplementary file 2 — Appendix S2. Modified Downs and Black Scoring Criteria [file JCSM-15-513-s005.docx]

**Appendix 2: Modified Downs and Black Scoring Criteria**

| **Number** | **Criteria** | **Scoring Criteria** | **Score** |
| --- | --- | --- | --- |
| 1 | Is the hypothesis/aim/objective of the study clearly described? | A point was given if the hypothesis aim or objective of the study was implicitly or explicitly indicated anywhere in the article. | 0 = No 1 = Yes |
| 2 | Are the main outcomes to be measured clearly described in the "Introduction" or "Methods" section? | A point was given if the main outcomes to be measured were clearly described in the "Introduction" or "Methods" section. | 0 = No 1 = Yes |
| 3 | Are the characteristics of the patients included in the study clearly described? | A point was given if the inclusion or exclusion criteria, or both, were indicated. | 0 = No 1 = Yes |
| 6 | Are the main findings of the study clearly described | A point was not awarded if the main outcome is not clearly addressed in the results or if the significance of results could not be interpreted. | 0 = No  1 = Yes |
| 7 | Does the study provide estimates of the random variability in the data for the main outcomes? | A point was awarded if the interquartile range (for non-normally distributed data), standard error, standard deviation, or confidence intervals (for normally distributed data) were reported. If the distribution of the data was not described, we assumed that the estimates used were appropriate, and we answered "yes" (1 point). | 0 = No 1 = Yes  |
| 10 | Have actual probability values been reported (e.g. 0.035 rather than < 0.05) for the main outcomes except where the probability value is less than 0.001? | A point was awarded if the exact P value was provided for both statistically significant and non-significant results for at least the main outcome measures. A point was not awarded if a study simply indicated that the results for the main outcome measures were not significant without providing the exact P value. | 0 = No 1 = Yes |
|  | **EXTERNAL VALIDITY** |  |  |
| 11 | Were the subjects asked to participate in the study representative of the entire population from which they were recruited? | A point was awarded if the study identified the source population for patients and described how the patients were selected. Patients were determined to be representative if they comprised the entire source population, an unselected sample of consecutive patients, or a random sample (only feasible where a list of all members of the relevant population exists). Where a study did not report the proportion of the source population from which the patients are derived, the question was answered as unable to determine. | 1 = Yes 0 = No 0 = Unable to determine |
| 12 | Were those subjects who were prepared to participate representative of the entire population from which they were recruited? | The proportion of patients included in the study were representative of the population. Those asked who agreed to participate or responded should be stated. Validation that the sample was representative would include demonstrating that the distribution of the main confounding factors was the same in the study sample and the source population. No point was awarded if the proportion of those asked who agreed to participate or responded was not stated. | 1 = Yes 0 = No 0 = Unable to determine |
|  | **INTERNAL VALIDITY - BIAS** |  |  |
| 18 | Were the statistical tests used to assess the main outcomes appropriate? | If the distribution of the data (normal or not) was not described, it was assumed that the estimates used were appropriate, and a point was awarded. No point was awarded for studies that reported qualitative or quantitative data without any form of statistical comparisons or if the statistical tests reported were not appropriate. | 1 = Yes 0 = No 0 = Unable to determine |
| 20 | Were the main outcomes measures used accurate (valid and reliable)? | A point was awarded if the primary outcome measures were thought to be valid and reliable (e.g., number of physical therapy visits per chart report), regardless of whether reliability or validity was reported. A point was not awarded if at least one of the primary outcome measures in the study was not valid or reliable or if this information was not reported or could not be determined (i.e., a questionnaire without reported validity or reliability). | 1 = Yes 0 = No 0 = Unable to determine |
