# Supplementary material for: Appetite and dietary intake endpoints in cancer cachexia clinical trials: Systematic Review 2 of the cachexia endpoints series
Source: J Cachexia Sarcopenia Muscle. 2024 Feb 11;15(2):513–35. doi: 10.1002/jcsm.13434 (PMC10995275; doi:10.1002/jcsm.13434)
Supplement: Supplementary file 3 — Table S1. Raw values of appetite scores pre‐ and posttreatment with delta, significance levels and effect sizes of two‐armed trials [file JCSM-15-513-s002.docx]

**Supplementary Table 1: Raw values of appetite scores pre- and posttreatment with delta, significance levels and effect sizes of two-armed trials**

| **Author (year)** | **Intervention period** | **Intervention** | **Control Baseline (mean ± SD)** | **Control Post treatment (mean ± SD)** | **Control ∆ (baseline – post treatment)** | **Intervention Baseline (mean ± SD)** | **Intervention Post treatment (mean ± SD)** | **Intervention ∆ (baseline - post** | **Effect size between arms (Hedge’s g)** | **p-value**  **between arms** | **Role of nutrition endpoint** |
| --- | --- | --- | --- | --- | --- | --- | --- | --- | --- | --- | --- |
| **VAS/NRS** | | | | | | | | | | | |
| **Famil-Dardashti *et al.* (2020) [93]** | 8 Weeks | Herbal combination (Fenugreek, Fennel, Chicory) + MA | 2.8 ± 0.4 | 3.0 ± 0.04 | - | 4.9 ± 0.6 | 3.9 ± 0.5 | - |  | 0.01 | Exploratory |
| **Ko *et al.* (2021) [101]** | 4 Weeks | Yukgunja-Tang + nutritional counseling | - | - | - | - | - | - |  | 0.058 | Secondary |
| **Maccio *et al.* (2011) [64]** | 16 Weeks | Lipoic acid + Carbocysteine + L-carnitine + Celecoxib + MA | 5.1 ± 1.6 | 6.3 ± 1.5 | - | 4.5 ± 1.5 | 6.0 ± 1.0 | - |  | 0.774 | Secondary |
| **McMillan *et al.* (1999) [41]** | 12 Weeks | MA + Ibuprofen | 3 (0-10) * | - | 1.0 (-3.0-9.2) * | 4 (0.3-9.1) * | - | 1.0 (-5.0-8.1) * |  | n.s | Exploratory |
| **Kouchaki *et al.* (2018) [83]** | 2 months | MA + celecoxib | 5.1 ± 1.6 | 8.2 ± 1.3 | - | 4.7 ± 1.6 | 7.2 ± 1.5 | - |  | 0.047 | Secondary |
| **Bruera *et al.* (2003) [47]** | 2 Weeks | Fish oil capsules (EPA 180mg, DHA, Vitamin E) | 6.65 ± 1.9 | 5.75 ± 2.4 | -0.9 ± 2.7 | 5.75 ± 2.7 | 4.77 ± 2.0 | -0.98 ± 2.0 | -0.034 | n.s | Primary |
| **De Conno *et al.* (1998) [38]** | 2 Weeks | MA | - | - | 0 (-1.0-2.5)* | - | - | 3 (2.0-4.0)* |  | 0.0064 | Primary |
| **Del Fabro *et al.* (2013) [66]** | 4 Weeks | Melatonin | 6 (4-9) * | - | -1.19 ± 2.3 | 7 (6-8) * | - | -0.83 ± 2.6 | 0.147 | 0.8 | Primary |
| **Hunter *et al.* (2021) [99]*** | 4 Weeks | Mirtazapine | 3 (2-4) * | 2 (0-2) * | - | 3 (2-5) * | 2 (0-2) * | - |  | 0.472 | Primary |
| **Navari and Brenner (2010) [60]** | 8 Weeks | MA 800 mg/day + Olanzapine 5 mg/day | 1.7 ± 2.1 | 2.0 ± 2.3 | - | 1.5 ± 1.9 | 6.3 ± 3.7 | - |  | - | Primary |
| **Dobrila-Dintinjana *et al.*  (2013) [67]** | 5 years | Counseling incl. MA and EPA-enriched ONS | - | - | 43.18** | - | - | -7.8** |  | - |  |
| **Laviano *et al.,* (2020) [89]** | 12 weeks | N-3 PUFAs (2g), 25-hydroxy-vitamin D3 and high-qual-ity whey protein | - | - | - | - | - | - |  | n.s | Secondary |
| **Ziętarska *et al.,* (2017) [81]** | NA | Nutritional support with high protein (ONS) | 6.40 ± 2.00 | 5.90 ± 2.22 | - | 6.44 ± 2.68 | 7.79 ± 1.7 | - |  | 0.0001 | Secondary |
| **Downer *et al.* (1993) [28]** | 6 Weeks | Medroxyprogesterone acetate (MPA) | 2.44 | 3.20 | - | 2.06 | 5.09 | - |  | - | Primary |
| **Wen *et al.* (2012) [63]** | 8 Weeks | MA + Thalidomide | 4.6 ± 1.4 | 5.5 ± 1.9 | - | 4.5 ± 1.5 | 5.6 ± 2.0 | - |  | 0.12 | Secondary |
| **Feliu *et al.*  *(*1992) [27]** | 2 Months | Counseling incl. MA and EPA-enriched ONS | - | - | 13.1*** | - | - | 48.4*** |  | <0.05 | Secondary |
| **Simons *et al.* (1996) [35]** | 12 Weeks | MPA | 6.0 ± 2.9 |  | -0.6 ± 2.4 | 5.8 ± 2.9 | - | +0.8 ± 2.7 | 0.545 | 0.01 | Primary (multiple) |
| **Turcott *et al.* (2018) [85]** | 8 Weeks | Nabilone | 6.5 ± 2.08 | 5.3 ± 3.04 | -1.1 ± 3.7 | 9 ± 1.6 | 6.1 ± 3.1 | -2.8 ± 2.3 | -0.533 | 0.219 | Exploratory |
| **Madeddu *et al.* (2012) [65]** | 4 Months | L-carnitine + Celecoxib + MA | 6.2 ± 2.3 | 7.6 ± 2.8 | - | 5.9 ± 1.8 | 7.3 ± 2.3 | - |  | 0.25 | Secondary/Exploratory |
| **Izumi *et al.* (2021) [100]** | 12 Weeks | Testosterone | 3.3 | 3.5 | - | 2.6 | 3.1 | - |  | n.s | Primary |
| **EORTC-C30/PAL15** | | | | | | | | | | | |
| **McMillan *et al.* (1999) [41]** | 12 Weeks | MA + Ibuprofen | 66.7 (0-100) * | 69.7 (-2-9) * | - | - | - | - |  | - | Exploratory |
| **Kouchaki *et al.,* (2018) [83]** | 2 months | MA + Celecoxib | 5.1 ± 1.6 | 6.9 ± 1.5 | - | 4.7 ± 1.6 | 6.4 ± 1.4 | - |  | 0.111 | Secondary |
| **Britton *et al.* (2018) [87]** | 12 Weeks | Motivational interview and cognitive behavioral therapy | - | - | - | - | - | - |  | 0.02 | QoL secondary |
| **Poulsen et al. (2014) [71]** | 5/12 weeks (depending on treatment + diagnosis) | Nutritional counseling + High-protein nutrition supplement incl EPA (2.2g) | - | - | - | - | - | - |  | <0.05 | QoL secondary |
| **Focan *et al.* (2015) [72]** | 2 months | Dietetic and psychological mindfulness workshops | - | - | - | - | - | - |  | n.s | QoL primary (one of several) |
| **Persson *et al.*  (2009) [45]** | 24 Months | 1) Individual support (IS), including nutritional and psychological support 2) IS + GR | 30 ± 34 | <4 ± 13 | - | 32 ± 35 | <4 ± 13 | - |  | n.s | One of several QoL endpoints |
| **Silander *et al.*  (2012) [62]** | 2 years | Tube feeding | 24 | 12 | - | 20 | 15 | - |  | n.s | One of several QoL endpoints |
| **Kutz *et al.,* (2022) [103]** | Oct 2018- Oct 2020 | Individualised nutritional counselling | 0 (50) * | 66.7 (66.7) * | - | 0 (33.3) * | 66.6 (66.7) * | - |  | n.s | QoL primary |
| **Pottel *et al.* (2014) [70]** | May 2012 – Dec 2013 | Echium oil 7.5 ml twice a day during treatment (Omega-3) | - | - | - | - | - | - |  | n.s | QoL secondary |
| **Qui *et al.* (2020) [96]** | 6 Weeks | Whole course nutrition management | 12.49 ± 17.99 | 20.83 ± 14.63 | -- | 13.33 ± 20.59 | 15.55 ± 8.41 | - |  | 0.023 | One of several QoL endpoints |
| **Westman *et al.* (1999) [42]** | 12 Weeks | MA | 46.8 ± 38.7 | - | - | 48.0 ± 36.1 | - | - |  | 0.19 | One of several QoL endpoints |
| **Woo *et al.* (2016) [76]** | 8 Weeks | Pancreatic exocrine replacement therapy | - | 57.69 ± 35.97 | - | - | 38.89 ± 35.11 | - |  | 0.05 | Exploratory |
| **Hong, Wu and Wu (2020) [94]** | 12 Weeks | Resistance exercise | 17.6 ± 7.5 | 22.6 ± 6.9 | - | 18.3 ± 8.9 | 12.2 ± 7.1 | - |  | 0.012 | Secondary |
| **Schink *et al.* (2018) [84]** | 12 Weeks | Exercise + Counselling | 40.72 ± 37.22 | 24.06 ± 32.00 | -16.67 ± 46.45 | 21.20 ± 31.06 | 15.72 ± 28.59 | -5.47 ± 27.81 | 0.345 | 0.051 | QoL Secondary |
| **Storck *et al.,* (2020) [97]** | 12 weeks | Physical excercise and whey protein supplement with excercise | - | - | - | - | - | - |  | n.s | QoL secondary |
| **Uster *et al.* (2019) [86]** | 6 Months | Counselling + exercise |  |  | 5.9 ± 7.8 (SE) |  |  | 6.5 ± 6.7 (SE) | 0,019 | 0.14 | QoL Primary |
| **Bouleuc *et al.* (2020) [91]*** | Until death (median follow up 33.8 months) | Parenteral nutrition | - | 2.46 (1.91-3.65) | - | - | 1.45 (1.05-2.79) | - |  | 0.233 | Secondary |
| **Dehghani *et al.* (2020) [92]** | 2 Months | Captopril | 50.0 ± 31.5 | 33.3 ± 26.4 | - | 50.0 ± 35.1 | 35.0 ± 33.2 | - |  | n.s | QoL Primary |
| **Simons *et al.* (1996) [35]** | 6 weeks | MPA | 29.1 ± 37.1 | - | +6.1 ± 33.9 | 35.7 ± 37.9 | - | -6.2 ± 39.9 | -0,390 | 0.06 | QoL secondary |
| **Turcott *et al.* (2018) [85]** | 8 Weeks | Nabilone | 76.6 ± 22.4 | 49.9 ± 45.1 | - | 92.5 ± 22.2 | 62.9 ± 30.9 | - |  | - | Exploratory |
| **Werner *et al.,* (2017) [80]** | Feb 2011 – Feb 2011 | Capsules with marine omega-3 fatty acids as phospholipids. 6.9% EPA and 13.6 % DHA. | - | - | - | -- | - | - |  | n.s | QoL secondary |
| **QoL-ACD** | | | | | | | | | | | |
| **Katakami *et al.* (2018) [82]** | 12 Weeks | Anamorelin | - | - | 0.3 ± 0.1 (SE) | - | - | 0.7 ± 0.1 (SE) | 0,444 | 0.0005 | QoL secondary |
| **NCCTG** (Change recorded as % of appetite improvement)** | | | | | | | | | | | |
| **Jatoi *et al.* (2007) [56]** | 24 Weeks | Etanercept | - | - | 44 | - | - | 57 |  | 0.31 | Secondary |
| **Jatoi *et al.* (2016) [74]** | 3-4 Weeks | White wine | - | - | 36 | - | - | 31 |  | 0.01 | Primary |
| **Jatoi *et al.* (2010) [58]** | Mean number of days on study 68 | Infliximab | - | - | - | - | - | - |  | n.s | Secondary |
| **Jatoi *et al.* (2004) [49]** | 3 months | EPA | - | - | 68 | - | - | 74 |  | 0.59 | Secondary |
| **Jatoi (2017) [77]** | 1 Month | Creatine | - | - | 8 | - | - | 12 |  | 0.49 | Exploratory |
| **Kardinal *et al.* (1990) [25]** | Median time in study 34 days cyproheptadine, 39 days placebo | Cyproheptadine | - | - | 36 | - | - | 55 |  | 0.01 | Secondary |
| **Loprinzi *et al.* (1990) [26]** | Median time on study 1.6 months | MA | - | - | 39 | - | - | 63 |  | 0.003 | Secondary |
| **Goldberg *et al.* (1995) [32]** | Treatment continued until appeared suggestion of benefit by patient/physician (aim to continue until loss of ≥ 5% of study body weight) | Pentoxifylline | - | - | 46 | - | - | 28 |  | 0.16 | Secondary |
| **Symptom distress scale** | | | | | | | | | | | |
| **Gebbia, Testa and Gebbia (1996) [34]** | 30 days | MA | 3.1 | - | - | 2.9 | - | - |  | 0.305 | One of several (order not defined) |
| **Ulutin, Arpaci and Pak (2002) [46]** | 3 months | MA 160mg/day | 2.8 | - | - | 2.9 | - | - |  | 0.28 | One of several (order not defined) |
| **FAACT ACS** | | | | | | | | | | | |
| **Jatoi *et al.* (2004) [49]*** | 4 Weeks | Eicosapentaenoic acid (EPA) | 55 (28-79) | 55 | - | 55 (30-80) | 40 | - |  | 0.004 | Secondary |
| **HCP** | | | | | | | | | | | |
| **Erkurt, Erkisi and Tunali, (2000) [43]** |  |  |  |  |  |  |  |  |  |  |  |

DHA Docosahexaenoic acid, EPA Eicosapentaenoic acid, MA Megesterol Acetate, MPA Medroxyprogesterone Acetate, ONS Oral Nutritional Supplement, PUFA Polyunsaturated Fatty Acids

*Results recorded as median

** (Change recorded as % of appetite improvement)

***Difference in loss of appetite from baseline – post treatment
