# Supplementary material for: Appetite and dietary intake endpoints in cancer cachexia clinical trials: Systematic Review 2 of the cachexia endpoints series
Source: J Cachexia Sarcopenia Muscle. 2024 Feb 11;15(2):513–35. doi: 10.1002/jcsm.13434 (PMC10995275; doi:10.1002/jcsm.13434)
Supplement: Supplementary file 4 — Table S2. Raw values of appetite scores pre‐ and posttreatment with delta, significance levels and effect sizes of multi‐armed trials [file JCSM-15-513-s004.docx]

**Supplementary Table 2: Raw values of appetite scores pre- and posttreatment with delta, significance levels and effect sizes of multi-armed trials**

| **Author (year)** | Intervention period | Interventions | Arm 1 ∆ from baseline | Arm 2 ∆ from baseline | Arm 3 ∆ from baseline | Role of nutrition endpoint (Primary, secondary, exploratory) |
| --- | --- | --- | --- | --- | --- | --- |
| **VAS/NRS** | | | | | | |
| **Catalina *et al.* (1998) [37]** | 12 weeks | ARM1; Placebo, ARM2; 160mg MA, ARM3; 480mg MA | 1.99 | 3.04 | 3.47 | Exploratory |
| **Kanat et al. (2013) [68]** | 3 months | ARM1; MA+ Meloxicam, ARM2; MA + meloxicam + +EPA-riched supplement ARM3; meloxicam + oral EPA-enriched nutritional supplement |  | - | - | Secondary |
| **Currow et al. (2021) [23]** | 1 week | ARM1; Placebo, ARM2; MA, ARM3; Dexamethasone | - | 2.68 (∆ in comparison to placebo) | 1.34 (∆ in comparison to placebo) | Primary |
| **Strasser et al. (2006) [55]** | 6 weeks | ARM1; Placebo, ARM2; Cannabis extract, ARM3; delta-9-tetrahydrocannabinol | 5.8 (± 23.8) | 5.4 (± 24.7) | 0.6 (± 18.5) | Primary |
| **Beller et al. (1997) [36]** | 12 weeks | ARM1; Placebo, ARM2; 160mg MA, ARM3; 480mg MA | 9.7 | 17.0 | 31.3 | Secondary |
| **Mantovani et al. (2010) [59]***** | 4 months | ARM1; MPA 500mg/day or MA 320mg/day, ARM 2; ONS with EPA, ARM3; L-carnitine, ARM4; Thalidomide, ARM5; combination of above | 1.4 | -0.5 | 0.2 | Secondary |
| **EORTC-C30** | | | | | | |
| **Fearon et al. (2006) [54]** | 8 weeks | ARM1; Placebo, ARM2; EPA 2g, ARM3; EPA 4g | - | -6.6 (comparison with placebo) | -1.2 (comparison with placebo) | Exploratory |
| **Baldwin et al. (2011) [61]** | 8 weeks | ARM1; Placebo, ARM2; EPA 2g, ARM3; EPA 4g | - | - | - | Exploratory |
| **Ravasco et al. (2005) [53]** | 3 months | ARM1; Ad-lib intake (G3), ARM2; dietary counseling (G1), ARM3; supplements (G2) | 23 | 3 | 32 | One of several QoL end points |
| **QoL ACD** | | | | | | |
| **Takayama et al. (2016) [75]** | 12 weeks | ARM1; Placebo, ARM2; 50mg anamorelin, ARM3; 100mg anamorelin | - | - | - | Secondary |
| **NCCTG** | | | | | | |
| **Loprinzi *et al.* (1993) [29]** | Median follow up 66 days | ARM1; MA 160mg, ARM2; MA 800mg, ARM3; MA 1280mg | - | - | - | Primary |
| **Jatoi (2002) [44]** | As long as patients/healthcare providers thought treatment beneficial or until toxic side effects | ARM1; oral MA, ARM2; oral dronabinol + placebo, ARM3; MA + dronabinol | 16* | 8* | 15* | Primary |
| **Loprinzi et al. (1999) [40]** | 1 month | ARM1; MA, ARM2; dexamethasone, ARM3; fluoxy-mesterone | 74* | 66* | 60* | Primary |
| **HCP** | | | | | | |
| **Chen et al. (1996) [33]** | 8 weeks | ARM1; Control, ARM2; megace, ARM3; cisapride | 3.99 ± 5.52 | -1.71 ± 4.56 | -5.41 ± 3.19 | One of several (not ordered) |
| **Study specific** | | | | | | |
| **Lai, Fang and Yeh (1994) [31]** | 21 days | ARM1; Placebo, ARM2; MA, ARM3; Prednisolone | 4** | 11** | 6** | Secondary |

MA Megesterol Acetate, MPA Medroxyprogesterone Acetate, ONS Oral Nutritional Supplement

*Change recorded as percentage of improvement

** Number of patients with appetite improvement

*** 5 arm study, Arm 4 ∆ from baseline 0.3, Arm 5 ∆ from baseline 1.0
