# Supplementary material for: Appetite and dietary intake endpoints in cancer cachexia clinical trials: Systematic Review 2 of the cachexia endpoints series
Source: J Cachexia Sarcopenia Muscle. 2024 Feb 11;15(2):513–35. doi: 10.1002/jcsm.13434 (PMC10995275; doi:10.1002/jcsm.13434)
Supplement: Supplementary file 5 — Table S3. Raw values of dietary intake of energy and protein pre‐ and posttreatment with delta, significance levels and effect sizes of two‐armed trials [file JCSM-15-513-s006.docx]

**Supplementary Table 3: Raw values of dietary intake of energy and protein pre- and posttreatment with delta, significance levels and effect sizes of two-armed trials**

| **Author (year)** | **Intervention period** | **Intervention** | **Control Baseline (mean ± SD)** | **Control Post treatment (mean ± SD)** | **Control ∆ (baseline – post treatment)** | **Intervention Baseline (mean ± SD)** | **Intervention Post treatment (mean ± SD)** | **Intervention ∆ (baseline – post)** | **Effect size** | **p-value**  **between arms** | **Role of nutrition end-point** |
| --- | --- | --- | --- | --- | --- | --- | --- | --- | --- | --- | --- |
| **Food records (energy)** | | | | | | | | | | | |
| Cereda^a)^ *et al.,* (2019) [88] | 3 months | Whey protein isolate supplementation | - | - | - | - | - | - | - | - | Exploratory |
| Fearon *et al., (*2003) [48] | 8 weeks | n-3 fatty acid enriched oral supplement | 1613 ± 51^b)^ kcal/day | - | +68 ± 64^b)^ kcal/day | 1504 ± 54^b)^ kcal/day | - | +224 ± 68^b)^ kcal/day | 0,33 | ns | Not clear |
| Bargetzi *et al.,* (2021) [98] | 30 days | Individualised nutritional support | - | 16.6 ± 9.0 kcal/kg/d | - | - | 20.9 ± 10.4 kcal/kg/d | - | - | <0.001 | Exploratory |
| Bourdel-Marchasson *et al.,* (2014) [69] | 3-6 months | Nutritional advice | - | - | +132kcal/day | - | - | +328 kcal/day | - | <0.01 | Secondary |
| Bruera *et al.,* (2003) [47] | 2 weeks | Fish oil | 4047 ± 1555 kcal | - | -57 ± 1299kcal | 4160 ± 1382 kcal | - | +51 ± 1177 kcal | 0,09 | ns | Secondary |
| Ovesen *et al.,* (1993) [30] | 5 months | Nutritional counselling | 1791.38 ± 549.36 kcal/day | - | -71.66 ± 477.7 kcal/day | 1839.16 ± 453.82 kcal/day | - | 143.31 ± 549.36 kcal/day | 0,42 | <0.05 | Exploratory |
| Bauer *et al.,* (2005) [51] | 8 weeks | Oral nutritional supplement | 1058 ± 112 kcal/day (or 18.13 ± 1.82 kcal/kg/day) | 1556 ± 74 kcal/day (26.96 ± 1.28kcal/kg/day) | - | 1576 ± 36 kcal/day (or 26.17 ± 0.61 kcal/kg/day) | 2037 ± 63 kcal/day (33.11 kcal/kg/day) | - | - | <0.001 | Primary |
| Beijer *et al.,* (2009) [57] | 8 weeks | Adenosine 5’-triphosphate infusions | 7.4 ± 2.1 MJ | 7.7 ± 0.4MJ | - | 6.8 ± 2.1 MJ | 7.1 ± 0.4 | - | - | 0.74 | Primary |
| Lundholm *et al.,* (2004) [50] | Until death | Indomethacin, Erythropoietin and nutritional support | 1774 ± 49kcal/day | 1695 ± 77 kcal/day | - | 1686 ± 56kcal/day | 2365 ± 102 kcal/day | - | - | 0.03 | Primary |
| Woo *et al.,* (2016) [76] | 8 weeks | Pancreatic exocrine replacement therapy | - | 1297.7 ± 552.8 | - | - | 1487.5 ± 6552 | - | - | 0.29 | Secondary |
| Stork *et al.,* (2020) [97] | 12 weeks | Leucine-rich supplement + nutrition + exercise | 85.4 ± 24.8 % | - | -11.31% (3 months) | 87.8 ± 24.4 % | - | -0.73% (3 months) | - | 0.207 | Secondary |
| Uster *et al.,* (2018) [86] | 3 months | Nutritional counselling and exercise program | - | - | -11 ± 130kcal/day | - | - | +129 ± 6 kcal/day | - | 0.17 | Secondary |
| Molassiotis *et al.,* (2021) [102] | 4-6 weeks | Family centred psychosocial-based nutrition intervention | 932.14 ±88.40 kcal | 876.69 ± 99.35 kcal | -43.49 ± 68.99 kcal | 1078.58 ± 102.51kcal | 1255.76 ± 106.45 kcal | +182.81 ±126.06 kcal | 0,78 | 0.77 | Co-primary |
| **Food records (protein)** | | | | | | | | | | | |
| Cereda^a)^ *et al.,* (2019) [88] | 3 months | Whey protein isolate supplementation | - | - | - | - | - | - | - | - | Exploratory |
| Fearon *et al., (*2003) [48] | 8 weeks | n-3 fatty acid enriched oral supplement | 63 ± 2g/day | - | 6 ± 3.3g | 60 ± 2g/day | - | 15 ± 3.5g | 0,37 | ns | Not clear |
| Bargetzi *et al.,* (2021) [98] | 30 days | Individualised nutritional support | - | Mean protein intake 0.6 ± 0.4g/kg/day | - | - | Mean protein intake 0.8g/kg/day | - | - | <0.001 | Exploratory |
| Bourdel-Marchasson *et al.,* (2014) [69] | 3-6 months | Nutritional advice | - | - | - | - | - | - | - | - | Secondary |
| Bruera *et al.,* (2003) [47] | 2 weeks | Fish oil | - | - | - | - | - | - | - | - | No protein data |
| Ovesen *et al.,* (1993) [30] | 5 months | Nutritional counselling | 58 ± 19 g/day (or 0.9 ± 0.3g/kg/day) | - | -1 ± 22 g/day | 61 ± 18 g/day (or 0.9 ± 0.3g/kg/day) | - | 8 ± 21g/day | 0,42 | <0.05 | Exploratory |
| Bauer *et al.,* (2005) [51] | 8 weeks | Oral nutritional supplement | 36.6 ± 4.7g/day (or 0.67 ± 0.08g/kg/day) | 62.4 ± 3.5 g/day (or 1.08 ± 0.06g/kg/day) | - | 62.3 ± 1.7g/day (or 1.04g/kg/day) | 87.2 ± 2.7 g/day (1.43 ± 0.04g/kg/day) | - | - | <0.001 | Primary |
| Beijer *et al.,* (2009) [57] | 8 weeks | Adenosine 5’-triphosphate infusions | 65 ± 4g/day | 69 ± 5g/day | - | 60 ± 3g/day | 65 ± 5g/day | - | - | 0.52 | Primary |
| Lundholm *et al.,* (2004) [50] | Until death | Indomethacin, Erythropoietin and nutritional support | - | - | - | - | - | - | - | - | No protein data |
| Woo *et al.,* (2016) [76] | 8 weeks | Pancreatic exocrine replacement therapy | - | 53.85 ± 25.23g/day | - | - | 56.72g/day | - | - | 0.7 | Secondary |
| Stork *et al.,* (2020) [97] | 12 weeks | Leucine-rich supplement + nutrition + exercise | 97.9 ± 37.9% | - | -6.43% | 104.4 ± 31.9% | - | 8.24% | - | 0.094 | Secondary |
| Uster *et al.,* (2018) [86] | 3 months | Nutritional counselling and exercise program | - | - | -2 ± 5g/day^c)^ (at 3 months) | - | - | 6 ± 4g/day^c)^ (at 3 months) | - | 0.01 | Secondary |
| Molassiotis *et al.,* (2021) [102] | 4-6 weeks | Family centred psychosocial-based nutrition intervention | 35.36 ± 3.46g | 34.90 ± 3.79g | 3.24 ± 2.22g | 36.93 ± 3.98g | 44.46 ± 4.09gl | 7.59 ± 3.72g | 0,49 | 0.76 | Co-primary |
| **24 h recall (energy)** | | | | | | | | | | | |
| Cereda^c)^ *et al.,* (2019) [88] | 3 months | Whey protein isolate supplementation | - | - | - | - | - | - | - | - | Secondary |
| Obling *et al.,* (2019) [90] | 12 weeks | Parenteral nutrition | 131.7 (92.6-239) kJ/kg/d^d)^ | 153.3 (91.6-228.5) kJ/kg/d ^d)^ | - | 158.8 (24.6-249.5) kJ/kg/day ^d)^ | 130.9 (59.9-320.9) kJ/kg/day ^d)^ | - | - | ns | Exploratory |
| Movahed *et al.,* (2020) [95] | Mean 44 ± 13 days | Individualized nutritional plan based on needs (energy 25-30 kcal/kg/day and protein 1.1-1.2 g/kg/day). ONS when needs were not met | 16.3 ± 9.4 Kcal/kg/d | 18.2 ± 11.2 Kcal/kg/d | 1.2 ± 14.4 Kcal/kg/d | 17.4 ± 9.6 Kcal/kg/d | 26.4 ± 14.1 Kcal/kg/d | 8.8 ± 15.4 | 0,51 | 0.003 | Secondary |
| Kapoor *et al.,* (2007) [78] | 6 months | Improved Atta (IAtta) | 756.7 ± 364.2 kcal | 803.0 ± 525.0 kcal | - | 947.4 ± 327.9 kcal | 1485.3 ± 477.4 kcal | - | - | 0.001 | Secondary |
| **24h recall (protein)** | | | | | | | | | | | |
| Cereda ^c)^ *et al.,* (2019) [88] | 3 months | Whey protein isolate supplementation | - | - | - | - | - | - | - | - | Secondary |
| Obling *et al.,* (2019) [90] | 12 weeks | Parenteral nutrition | 1.11 (0.33-2.32) g/kg/d^d)^ | 1.10 (0.69-1.69) g/kg/d ^d)^ | - | 1.18 (0.13-1.76) g/kg/d ^d)^ | 1.12 (0.45-2.29) g/kg/d ^d)^ | - |  | <0.05 | Exploratory |
| Movahed *et al.,* (2020) [95] | Mean 44 ± 13 days | Individualized nutritional plan based on needs (energy 25-30 kcal/kg/day and protein 1.1-1.2 g/kg/day). ONS when needs were not met | 0.5 ± 0.4g/kg/d | 0.7 ± 0.4 g/kg/d | 0.1 ± 0.5 g/kg/d | 0.6 ± 0.4 g/kg/d | 1.0 ± 0.6 g/kg/d | 0.3 ± 0.7 g/kg/d | 0,33 | 0.005 | Secondary |
| Kapoor *et al.,* (2007) [78] | 6 months | Improved Atta (IAtta) | 23.2 ± 12.6 g | 23.2 ± 15.6g | - | 30.3 ± 12.0g | 49.5 ± 16.9g | - | - | <0.001 | Secondary |
| **Dietary history (energy)** | | | | | | | | | | | |
| Simons *et al.,* (1998) [39] | 12 weeks | Medroxyprogesterone acetate (MPA)  500 mg twice daily | 2025 ± 529 kcal/day | **-** | 0 kcal/day | 2320 ± 756 kcal/day | **-** | 418 kcal/day | - | 0.01 | Primary |
| Gavazzi *et al.,* (2016) [73] | 2 months | Enteral nutrition started post-op day 2. Given during night. Usual food during the day. | **-** | 30.2kcal/kg | - | - | 40.6kcal/kg | **-** | - | 0.0001 | Primary |
| Leedo *et al.,* (2017) [79] | 12 weeks | One main (413 kcal/serving) and ad libitum snack (168 kcal/serving) | 6,442 ± 2,926kJ/Day | **-** | **-** | 7,232 ± 3,112kJ/day | **-** | **-** | - | 0.35 | Secondary |
| **Dietary history (protein)** | | | | | | | | | | | |
| Simons *et al.,* (1998) [39] | 12 weeks | Medroxyprogesterone acetate (MPA)  500 mg twice daily | **-** | **-** | **-** | **-** | **-** | - | - | <0.05 | Primary |
| Gavazzi *et al.,* (2016) [73] | 2 months | Enteral nutrition started post-op day 2. Given during night. Usual food during the day. | Protein results not recorded | **-** | **-** | **-** | **-** | **-** | - | - | Primary |
| Leedo *et al.,* (2017) [79] | 12 weeks | One main (413 kcal/serving) and ad libitum snack (168 kcal/serving) | 55 ± 28.4 g/day | **-** | **-** | 60 ± 25.5 g/day | **-** | **-** | - | 0.38 | Secondary |

a) Cereda et al. used both food records and 24h recall, but it is unclear which of these methods that is reported b) Standard error of the mean c) Unclear if standard deviation or standard error d) Median (range)
