# Supplementary material for: Appetite and dietary intake endpoints in cancer cachexia clinical trials: Systematic Review 2 of the cachexia endpoints series
Source: J Cachexia Sarcopenia Muscle. 2024 Feb 11;15(2):513–35. doi: 10.1002/jcsm.13434 (PMC10995275; doi:10.1002/jcsm.13434)
Supplement: Supplementary file 6 — Table S4. Raw values of dietary intake of energy and protein pre‐ and posttreatment with delta, significance levels and effect sizes of multi‐armed trials [file JCSM-15-513-s001.docx]

**Supplementary Table 4: Raw values of dietary intake of energy and protein pre- and posttreatment with delta, significance levels and effect sizes of multi-armed trials**

| **Author (year)** | Intervention period | Interventions | Arm 1 ∆ from baseline | Arm 2 ∆ from baseline | Arm 3 ∆ from baseline | P value | Role of nutrition end point |
| --- | --- | --- | --- | --- | --- | --- | --- |
| **24-hour recall (energy)** | | | | | | | |
| Dias *et al.,* (2005) [52] | Mean 70 ± 3 days | Arm 1 Oral  Arm 2 Feeding tube  Arm 3 Supplement group | 279kcal | 996kcal | 92.6kcal | - | Primary |
| Ravasco^a)^ *et al.,* (2005) [53] | During and 3 months after radiotherapy | Arm 1 Dietary counselling  Arm 2 Supplements  Arm 3 Ad lib intake | - | - | - |  | Secondary |
| **24-hour recall (protein)** | | | | | | | |
| Dias *et al.,* (2005) [52] | Mean 70 ± 3 days | Arm 1 Oral  Arm 2 Feeding tube  Arm 3 Supplement group | 19.4g | 41.9g | 13g | - | Primary |
| Ravasco^a)^ *et al.,* (2005) [53] | During and 3 months after radiotherapy | Arm 1 Dietary counselling  Arm 2 Supplements  Arm 3 Ad lib intake | - | - | - |  | Secondary |
| **Dietary history (energy)** | | | | | | | |
| Ravasco^a)^ *et al.,* (2005) [53] | During and 3 months after radiotherapy | Arm 1 Dietary counselling  Arm 2 Supplements  Arm 3 Ad lib intake |  |  |  |  | Secondary |
| **Dietary history (protein)** | | | | | | | |
| Ravasco^a)^ *et al.,* (2005) [53] | During and 3 months after radiotherapy | Arm 1 Dietary counselling  Arm 2 Supplements  Arm 3 Ad lib intake |  |  |  |  | Secondary |

a) Ravasco et al. used two dietary intake methods, but it is unclear which of these methods that is reported
